# Monte Carlo Simulations Suggest Current Chlortetracycline Drug-Residue Based Withdrawal Periods Would Not Control Antimicrobial Resistance Dissemination from Feedlot to Slaughterhouse

**DOI:** 10.3389/fmicb.2017.01753

**Published:** 2017-09-20

**Authors:** Casey L. Cazer, Lucas Ducrot, Victoriya V. Volkova, Yrjö T. Gröhn

**Affiliations:** ^1^Department of Population Medicine and Diagnostic Sciences, College of Veterinary Medicine, Cornell University Ithaca, NY, United States; ^2^Department of Diagnostic Medicine/Pathobiology, Institute of Computational Comparative Medicine, College of Veterinary Medicine, Kansas State University Manhattan, KS, United States

**Keywords:** beef cattle, antibiotic resistance, enteric bacteria, food-borne pathogens, mathematical modeling, population pharmacokinetics, pharmacodynamics

## Abstract

Antimicrobial use in beef cattle can increase antimicrobial resistance prevalence in their enteric bacteria, including potential pathogens such as *Escherichia coli*. These bacteria can contaminate animal products at slaughterhouses and cause food-borne illness, which can be difficult to treat if it is due to antimicrobial resistant bacteria. One potential intervention to reduce the dissemination of resistant bacteria from feedlot to consumer is to impose a withdrawal period after antimicrobial use, similar to the current withdrawal period designed to prevent drug residues in edible animal meat. We investigated tetracycline resistance in generic *E. coli* in the bovine large intestine during and after antimicrobial treatment by building a mathematical model of oral chlortetracycline pharmacokinetics-pharmacodynamics and *E. coli* population dynamics. We tracked three *E. coli* subpopulations (susceptible, intermediate, and resistant) during and after treatment with each of three United States chlortetracycline indications (liver abscess reduction, disease control, disease treatment). We compared the proportion of resistant *E. coli* before antimicrobial use to that at several time points after treatment and found a greater proportion of resistant enteric *E. coli* after the current withdrawal periods than prior to treatment. In order for the proportion of resistant *E. coli* in the median beef steer to return to the pre-treatment level, withdrawal periods of 15 days after liver abscess reduction dosing (70 mg daily), 31 days after disease control dosing (350 mg daily), and 36 days after disease treatment dosing (22 mg/kg bodyweight for 5 days) are required in this model. These antimicrobial resistance withdrawal periods would be substantially longer than the current U.S. withdrawals of 0–2 days or Canadian withdrawals of 5–10 days. One published field study found similar time periods necessary to reduce the proportion of resistant *E. coli* following chlortetracycline disease treatment to those suggested by this model, but additional carefully designed field studies are necessary to confirm the model results. This model is limited to biological processes within the cattle and does not include resistance selection in the feedlot environment or co-selection of chlortetracycline resistance following other antimicrobial use.

## Introduction

In many developed countries, the average person has very little or no contact with livestock, yet there is undoubtedly a risk of infectious agent transmission between livestock and human populations through the food supply chain and environmental intermediaries. Antimicrobial-resistant pathogens are capable of moving between animal and human populations via these pathways. Bacterial gene sequencing and multi-locus sequence typing suggest that whole bacterium transfer has occurred between at least poultry meat and humans (Overdevest et al., 2011; Kluytmans et al., 2013) and plasmid restriction-enzyme typing shows horizontal gene transfer of resistance elements between animal and human strains (Yan et al., 2004). Additionally, a recent metagenomic study on human feces found a higher prevalence of resistance genes to antimicrobials used in both animals and humans than resistance to human-limited antimicrobials (Forslund et al., 2013).

Consumers are therefore concerned about antimicrobial use in livestock and the risk of antimicrobial resistance spreading from livestock to humans by the consumption of animal products. Companies such as McDonalds, Tyson Foods, and Chipotle have responded with public commitments to limit or eliminate antimicrobials used in livestock in their supply chains (Chipotle, 2013; Tyson Foods, 2015; McDonalds, 2016). The Food and Drug Administration recently limited the use of medically-important antimicrobials in livestock for production purposes (i.e., growth promotion) and required veterinary oversight for antimicrobial use (US Food and Drug Administration, 2012, 2013). However, it is unrealistic to think that antimicrobials can be completely phased out of animal agriculture. Antimicrobials will continue to play a vital role in keeping livestock healthy and producing animal products efficiently and sustainably for a growing human population.

We must therefore devise antimicrobial stewardship policies to reduce the risk of antimicrobial resistance spread from livestock to humans. One possibility is to modify withdrawal periods required after antimicrobial use in livestock to account for resistance dynamics (Volkova et al., 2016). Antimicrobials used in livestock are already subject to meat and milk withdrawal times to prevent violative levels of drug residues from entering the human food supply. These withdrawal periods vary by country and by antimicrobial indication, varying from 0 to 10 days for oral chlortetracycline (P.R. Vademecum^1^; Hoffmann La-Roche Inc., 1997; Canadian Food Inspection Agency, 2014; Australian Pesticides and Veterinary Medicines Authority, 2015; Zoetis, 2016; EU Directorate General Health Consumers, 2017; New Zealand Ministry for Primary Industries, 2017; UK Veterinary Medicines Directorate, 2017). “Resistance withdrawals” could be used to decrease the prevalence of resistant bacteria and genes in an animal's enteric microbiome before it is sent for slaughter, where enteric bacteria can contaminate meat products (De Filippis et al., 2013).

In order to investigate the efficacy and length of resistance withdrawal periods that would reduce the prevalence of resistance in the livestock enteric microbiota, we built a model of the pharmacokinetics-pharmacodynamics of chlortetracycline (CTC) fed to beef steers, and the population dynamics of their enteric generic *Escherichia coli*. Chlortetracycline is a commonly used antimicrobial on beef feedlots (United States Department of Agriculture, 2013), and tetracyclines were identified as a highly important antimicrobial class for human medicine by the FDA (US Food Drug Administration, 2003), making CTC a suitable candidate for evaluating the impact of antimicrobial resistance withdrawal periods.

## Methods

The model consists of 3 sub-models that were connected and parameterized for CTC and generic *E. coli* in beef cattle: a pharmacokinetic model for the concentrations of orally administered drugs in the gastrointestinal tract (Cazer et al., 2014), a bacterial population dynamics model, and a pharmacodynamic model (Ayscue et al., 2009; Volkova et al., 2012). The framework for connecting these models and using them to investigate resistance withdrawal periods has been described (Volkova et al., 2016). Each model is described briefly below. Model equations are listed in Table 1. Each parameter was considered to be either a constant or a random variable. Each simulation of the three connected models represents one realization of a treated animal, with the values of the random variables drawn from the animal population-level parameter distributions. The model was parameterized using data available from literature wherever possible. For a parameter with only two published estimates, a Uniform distribution was used with the estimates as the maximum and minimum. For a parameter with only one published estimate, a Uniform distribution with the maximum and minimum being ±25% of the estimate was assigned (Volkova et al., 2017). For a parameter with at least 3 published estimates, the parameter distribution was determined by fitting candidate distributions to the dataset of the published estimates of the parameter values, with PROC CAPABILITY in SAS® 9.4 software for Windows (SAS Institute Inc., Cary, NC, U.S.); the best-fit distribution for the parameter was selected using goodness-of-fit tests (Anderson-Darling and Kolmogorov-Smirnov) and visual examination of quantile-quantile plots. The parameter distributions used in the model simulations are listed in Table 2. (The data used to fit the parameter value distributions for the parameters with at least 3 published estimates are included in Supplementary Materials).

### Model structure

#### Pharmacokinetic model

Equations of the pharmacokinetic model are given in Table 1A. The daily dose of CTC was fed to a 300 kg steer in equal parts throughout a 12-h day, during each of the treatment days (Table 1A, Equations 1–4). The drug moved through the steer's gastrointestinal tract (Table 1A, Equations 5–7) at the rate of concentrates, determined from studies using isotope-labeled feedstuffs (Shaver et al., 1986; Zebeli et al., 2007). In the small intestine, some CTC was absorbed into plasma and from there distributed into tissues (Bradley et al., 1982), excreted into urine and via bile back into the small intestine (Eisner and Wulf, 1963) (Table 1A, Equations 6, 8, 9). In all gastrointestinal, plasma, and tissue compartments, CTC degraded abiotically (Eisner and Wulf, 1963) into compounds with greatly reduced antimicrobial activity (Halling-Sørensen et al., 2002).

**Table 1A:** Pharmacokinetic model equations.

**Equation number**	**Equation**	**Description**
1	$CTC_{f}=\{\begin{array}{cc} \text{nighttime} & 0\\ \text{daytime} & \frac{CTC_{f}}{12} \end{array}$	Ingestion of chlortetracycline (CTC) at dosage (*CTC*_*f*_) in equal parts over a 12 h daytime.
2	*CTC*_*f*_*ARLA*__ = 70	Antimicrobial Reduction of Liver Abscesses (ARLA) Dosage, 70 mg per head daily.
3	*CTC*_*f*_*ADC*__ = 350	Antimicrobial Disease Control (ADC) Dosage, 350 mg per head daily.
4	$CTC_{f_{ADT}}=\{\begin{array}{cc} 22\times bw & t<120\\ 0 & t\geq 120 \end{array}$	Antimicrobial Disease Treatment (ADT) Dosage, 22 mg per kg body weight (*bw*) daily for 5 days [6,600 mg per head daily for 300 kg steer]. *t*- time of treatment, hour.
5	$\frac{dCTC_{s}}{dt}=\overset{\text{ingestion with feed}}{\overset{︷}{CTC_{f}}}-\overset{\text{transit to small intestine}}{\overset{︷}{\gamma_{s}CTC_{s}}}-\overset{\text{degradation}}{\overset{︷}{\delta CTC_{s}}}$	Change in stomach CTC amount (*CTC*_*s*_) from ingested *CTC*_*f*_, due to rates of fractional flow out of stomach (γ_*s*_) and degradation (δ).
6	$\begin{array}{l} \frac{dCTC_{upper\_si}}{dt}=\overset{\text{transit from stomachs}}{\overset{︷}{\gamma_{s}CTC_{s}}}+\overset{\text{biliary in-flow}}{\overset{︷}{k_{e}E_{b}CTC_{p}}}\\ -\overset{\text{absorption to central circulation}}{\overset{︷}{k_{a}CTC_{upper\_si}}}-\overset{\text{transit to lower small intestine}}{\overset{︷}{\gamma_{upper\_si}CTC_{upper\_si}}}-\;\overset{\text{degradation}}{\overset{︷}{\delta CTC_{upper\_si}}} \end{array}$	Change in upper small intestine CTC amount (*CTC*_*upper*_*si*_) due to rates of inflow from stomach (γ_*s*_), absorption to plasma (*k_*a*_*), outflow from upper small intestine (γ_*upper*_*si*_), and degradation (δ), as well as amount (*E_*b*_*) and rate (*k_*e*_*) of inflow from bile.
7	$\frac{dCTC_{rest\_si}}{dt}=\overset{\text{transit from small intestine}}{\overset{︷}{\gamma_{upper\_si}CTC_{upper\_si}}}-\overset{\text{transit to large intestine}}{\overset{︷}{\gamma_{rest\_si}CTC_{rest\_si}}}-\overset{\text{degradation}}{\overset{︷}{\delta CTC_{rest\_si}}}$	Change in lower small intestine CTC amount (*CTC*_*rest*_*si*_) due to rates of inflow from upper small intestine (γ_*upper*_*si*_), outflow from lower small intestine (γ_*rest*_*si*_), and degradation (δ).
8	$\begin{array}{l} a)\;\frac{dCTC_{p}}{dt}=\overset{\text{absorption from upper small intestine}}{\overset{︷}{k_{a}CTC_{upper\_si}}}-\overset{\text{excretion in bile}}{\overset{︷}{k_{e}E_{b}CTC_{p}}}-\overset{\text{excretion in urine}}{\overset{︷}{k_{e}E_{u}CTC_{p}}}\\ -\;\overset{\text{to tissues}}{\overset{︷}{k_{pt}CTC_{p}}}+\overset{\text{from tissues}}{\overset{︷}{k_{tp}CTC_{t}}}-\overset{\text{degradation}}{\overset{︷}{\delta CTC_{p}}}\\ b)\;\frac{dCTC_{p\_conc}}{dt}=\frac{CTC_{p}}{V_{p}} \end{array}$	(a) Change in plasma CTC amount (*CTC*_*p*_) due to rates of absorption in the upper small intestine (*k_*a*_*), excretion (*k_*e*_*) [excreted fractions in bile (*E_*b*_*) and urine (*E_*u*_*)], distribution to (*k_*pt*_*) and from (*k_*tp*_*) tissues and degradation (δ) (b) Calculation of plasma CTC concentration with plasma volume (*V_*p*_*).
9	$\frac{dCTC_{t}}{dt}=\overset{\text{to tissues}}{\overset{︷}{k_{pt}CTC_{p}}}-\overset{\text{from tissues}}{\overset{︷}{k_{tp}CTC_{t}}}-\overset{\text{degradation}}{\overset{︷}{\delta CTCt}}$	Change in tissue CTC amount (*CTC*_*t*_) due to rates of distribution from (*k*_*pt*_) and to (*k*_*tp*_) plasma, and degradation (δ).
10	$a)\;\frac{dCTC_{li}}{dt}=\overset{\text{transit from small intestine}}{\overset{︷}{\gamma_{rest\_si}CTC_{rest\_si}}}-\overset{\text{transit to defecation}}{\overset{︷}{\gamma_{li}CTC_{li}}}-\overset{\text{degradation}}{\overset{︷}{\delta CTC_{li}}}$ $b)\;\frac{dCTC_{li\_conc}}{dt}=\frac{CTC_{li}}{V_{li}}\times \left(1-\overset{\text{sorption to digesta}}{\overset{︷}{\eta }}\right)$	(a) Change in large intestine CTC amount (*CTC*_*li*_) due to rates of inflow from the small intestine (γ_*rest*_*si*_), outflow in feces (γ_*li*_), and degradation (δ) (b) Calculation of large intestine antimicrobially active CTC concentration with large intestine contents volume (*V_*li*_*) and accounting for the fraction of CTC adsorbed to digesta (η).

#### *Escherichia coli* population model

The model of *E. coli* growth considered only free-living, luminal *E. coli* in the large intestine because the amount of colon mucosal-associated *E. coli* is 1-2 magnitudes less than the luminal bacteria (Krause et al., 2003; Laven et al., 2003; Volkova et al., 2012). The previous model of an *E. coli* population divided the bacteria into two subpopulations, resistant and susceptible (Volkova et al., 2012). We expanded the model to include *E. coli* intermediately susceptible to tetracyclines (Clinical and Laboratory Standards Institute, 2015). We assumed that tetracycline resistance genes were carried on conjugative elements (transposons or plasmids) (Speer et al., 1992). Each subpopulation of bacteria can expand or contract due to logistic growth until the total population reaches a carrying capacity (Table 1B, Equation 11). The bacterial subpopulations are also affected by fitness costs of resistance elements, inflow and outflow of *E. coli* from the large intestine (Table 1B, Equations 12, 13), and horizontal plasmid/transposon transfer among resistant, intermediate, and susceptible strains (Table 1B, Equation 14) (Volkova et al., 2012).

**Table 1B:** *Escherichia coli* population and pharmacodynamic model equations.

**Equation number**	**Equation**	**Description**
11	$G_{j}=\overset{\text{subpopulation growth or decay}}{\overset{︷}{r\left(1-\frac{N}{N_{max}}\right)N_{j}}}\times \overset{\text{resistance fitness cost}}{\overset{︷}{\left(1-\alpha_{j}\right)}}\times \overset{\text{pharmacodynamic effect}}{\overset{︷}{E_{j}}}$	Growth of *E. coli* population *j*^1^ (susceptible, intermediate or resistant) at rate *r*, limited by carrying capacity *N_*max*_*, with reductions in growth from plasmid fitness cost α_*j*_ and pharmacodynamic effect *E_*j*_*. *N* is the total number of *E. coli* in the large intestine; *N_*j*_* is the number of *E. coli* in subpopulation *j*.
12	*In*_*j*_ = *p*_*j*_λ_*in*_*N*	Inflow of *j*^1^ *E. coli* into the large intestine, ingested from the environment, feed, or water, where *p_*j*_* is the ingested proportion *j, λ_*in*_* is the inflow rate, and *N* is the total number of *E. coli* in the large intestine.
13	*Out*_*j*_ = λ_*out*_*N*_*j*_	Outflow of *j*^1^ *E. coli* from the large intestine where λ_*out*_ is the outflow rate, and *N_*j*_* is the number of *j*^1^ *E. coli* in the large intestine.
14	$\begin{array}{l} \text{a) }PT_{is}=\beta \frac{N_{s}N_{i}}{N}\\ \text{b) }PT_{rs}=\beta \frac{N_{s}N_{r}}{N}\\ \text{c) }PT_{ri}=\beta \frac{N_{i}N_{r}}{N} \end{array}$	Transfer of plasmids/transposons from (a) intermediate to susceptible, (b) resistant to susceptible, and (c) resistant to intermediate *E. coli*. β is the rate of plasmid transfer between two *j* populations of *E. coli* and *N_*j*_* is the number of *j*^1^ *E. coli* in the large intestine, and *N* is the total number of *E. coli* in the large intestine.
15	$E_{j}=E_{0}-\frac{E_{max}CTC_{li\_conc}Hj}{{\left(EC_{50_{j}}\right)}^{Hj}+CTC_{li\_conc}Hj}$	Pharmacodynamic effect (*E_*j*_*) on growth rate of *j*^1^ *E. coli*. *E_*0*_* is the growth rate multiplier in the absence of CTC, *E_*max*_* is the maximum pharmacodynamic effect, *EC_*50j*_* is the CTC concentration that produces 50% of *E_*max*_, H_*j*_* is the Hill coefficient, and **CTC*_*li*_*conc*_* is the concentration of CTC in the large intestine.
16	log_2_ (*EC_50_j__*) = −1.24+1.09 log_2_(*MIC*_*j*_)	Relationship between *EC_*50j*_* and the minimum inhibitory concentration (*MIC_*j*_)* of CTC (Ahmad et al., 2015b).
17	$\begin{array}{l} \text{a) }\frac{dN_{s}}{dt}=G_{s}-PT_{is}-PT_{rs}+In_{s}-Out_{s}\\ \text{b) }\frac{dN_{i}}{dt}=G_{i}+PT_{is}-PT_{ri}+In_{i}-Out_{i}\\ \text{c) }\frac{dN_{r}}{dt}=G_{r}+PT_{ri}+PT_{rs}+In_{r}-Out_{r} \end{array}$	Change in the number of (a) susceptible, (b) intermediate, and (c) resistant *E. coli* over time due to the population growth, plasmid/transposon transfer, and inflow and outflow.

1 j population refers to s (susceptible), i (intermediate resistance), or r (resistant)

#### Pharmacodynamic model

Tetracyclines are bacteriostatic antimicrobials at physiologically achievable concentrations that inhibit protein synthesis and suppress the growth of *Enterobacteriaceae* (Norcia et al., 1999). The pharmacodynamic model was based on a sigmoid *E*_*max*_ model in which the pharmacodynamic effect is a reduction of the bacterial population growth rate (Table 1B, Equation 15) (Mouton and Vinks, 2005; Goutelle et al., 2008; Volkova et al., 2012; Ahmad et al., 2015b; Wen et al., 2016).

It has been shown that susceptible isolates of *Enterobacteriaceae* exhibit some growth suppression when exposed to tetracycline concentrations below their MIC, whereas resistant isolates are more robust and not significantly growth-suppressed until the concentration of tetracycline equals or exceeds their MIC (Gullberg et al., 2011; Ahmad et al., 2015b). Additionally, the drug concentration-effect relationship (captured by the pharmacodynamic parameter Hill coefficient) changes depending on the isolate MIC in *E. coli* and other Gram-negative pathogens (Ahmad et al., 2015b; Wen et al., 2016). Therefore, different Hill coefficients were applied for the susceptible, intermediate, and resistant E. coli subpopulations. For tetracycline in *E. coli, EC*_*50*_ is a function of MIC (Ahmad et al., 2015a,b). MIC is routinely measured under aerobic laboratory conditions but the lumen of the large intestine is anaerobic. This change in environment has been shown to affect the MIC of *E. coli* (DeMars et al., 2016) so we applied an anaerobic penalty to the standard aerobic MIC to reflect this difference.

### Model parameterization

#### Chlortetracycline dosing

In the United States, the chlortetracycline label indications for beef cattle are the control of active *Anaplasma marginale* infection and shipping fever (bacterial pneumonia) caused by *Pasteurella species* (350 mg per head daily; “ADC”), reduction of incidence of liver abscesses (70 mg per head daily; “ARLA”), and treatment of *Escherichia coli* enteritis or *Pasteurella* pneumonia (22 mg per kg bodyweight per day for 5 days [6,600 mg per head daily for a 300 kg steer]; “ADT”) (Zoetis Aureomycin 50^2^; ChlorMax 50^3^; Zoetis, 2016). Chlortetracycline was previously labeled for growth promotion and increased feed efficiency but those label claims were removed as of January 2017 in response to the FDA's Guidance for Industry #213 (US Food and Drug Administration, 2013). However, the dosage that was used for growth promotion (70 mg per head daily) is still labeled for the reduction of liver abscesses. In the model, CTC was fed at ARLA or ADC dosage for 28 days or at ADT dosage for 5 days. The treatment period for ADT was considered to last an additional 3 days because CTC concentrations in the large intestine remained increased for 3 days after ADT ended.

Some brand-name CTC products sold in the U.S. have no required withdrawal period for any of the dosages (Zoetis Aureomycin 50^2^). Generic CTC products sold in the U.S. have a 0-day withdrawal following ARLA, 2-day withdrawal following ADC, and 1-day withdrawal following ADT (Zoetis ChlorMax 50). In the model, the 0-day withdrawal was interpreted to mean that cattle could ingest CTC until they were put on a truck for transportation to slaughter and we assumed that they were slaughtered 6 h later. The travel time to the slaughterhouse was assumed to be included in the longer withdrawal periods (1 or 2 days).

#### Pharmacokinetic model

The pharmacokinetic parameters were the same as in previous models for CTC (Cazer et al., 2014; Volkova et al., 2017) and are listed in Table 2A. In summary, sufficient data for fitting a distribution to the parameter values from literature were available for only the CTC degradation rate. The best-fit model for the unweighted, mean CTC degradation rate at physiological pH and temperature (Eisner and Wulf, 1963; Carlson and Mabury, 2006; Arikan, 2008; Dolliver et al., 2008; Arikan et al., 2009) was a Beta distribution (Anderson-Darling *P* > 0.250, Chi-square *P* = 0.052) (Volkova et al., 2017). Only one estimate for the fraction of CTC excreted in bile was available (Eisner and Wulf, 1963); hence, the Uniform distribution of the parameter values was assigned the boundaries ±25% of the estimate. In the absence of cattle-specific data, the fraction of CTC adsorbed to digesta was parameterized with data from the bioavailability of tetracycline in rat feces (Bahl et al., 2004) and protein-bound CTC in dog serum (Pindell et al., 1959); these estimates were used as the boundaries for the Uniform distribution of the parameter values. Finally, a Uniform distribution for the volume of the contents in the large intestine was estimated using the data on the content weights at slaughter (Murray et al., 1977) and the density of fresh cattle feces (Volkova et al., 2017) (Table 2A). The parameters related to the distribution of CTC in central circulation and in tissues were kept constant in order to focus on the variability of CTC concentration in the large intestine. For this modeling exercise, we assumed that the cattle ate a grain-based diet and the CTC flow rates through gastrointestinal compartments were parameterized accordingly (Shaver et al., 1986; Zebeli et al., 2007). Only single estimates were available for the transit rates of concentrates through the small intestine and large intestine (Shaver et al., 1986); hence, each of these transit rates was assigned a Uniform distribution with the boundaries ±25% of the published estimate. Two estimates for the transit rate from the stomachs to small intestine were located (Shaver et al., 1986; Zebeli et al., 2007) but there was only a 0.0005 h^−1^ difference between the estimates; hence, a Uniform distribution with the boundaries ±25% of the average of the two estimates was assigned to the stomachs' transit rate. The steer's body weight was kept constant since growth over 40 days was previously found to have only minor impacts on CTC concentration in the large intestine in this model (Cazer et al., 2014).

**Table 2a:** Pharmacokinetic model parameters.

**Parameter**	**Distribution**	**Unit**	**Definition**	**References**	**Realized parameter range**
δ	Beta (0.54, 37.4)	h^−1^	Abiotic degradation rate	Eisner and Wulf, 1963; Carlson and Mabury, 2006; Arikan, 2008; Dolliver et al., 2008; Arikan et al., 2009	7.0675e^−10^, 0.0071, 0.1797
γ_*s*_	Uniform (0.0535, 0.0895)	h^−1^	Fractional flow from stomachs to small intestine	Shaver et al., 1986; Zebeli et al., 2007	0.0535, 0.072, 0.0895
γ_*upper*_*si*_	Uniform (0.250, 0.416)	h^−1^	Fractional flow through the upper 1/3 small intestine	Shaver et al., 1986; Martin et al., 1999	0.2502, 0.3337, 0.416
γ_*rest*_*si*_	Uniform (0.100, 0.166)	h^−1^	Fractional flow through the lower 2/3 small intestine	Shaver et al., 1986; Martin et al., 1999	0.1, 0.1331, 0.166
γ_*li*_	Uniform (0.100, 0.166)	h^−1^	Fractional flow through large intestine	Shaver et al., 1986	0.1, 0.1334, 0.166
*k_*a*_*	0.0478 (Constant)	h^−1^	Absorption into plasma rate	Reinbold et al., 2010	–
*k_*pt*_*	0.7500 (Constant)	h^−1^	Distribution from plasma into tissues rate	Bradley et al., 1982	–
*k_*tp*_*	0.1620 (Constant)	h^−1^	Distribution from tissues into plasma rate	Bradley et al., 1982	–
*k_*e*_*	1.1400 (Constant)	h^−1^	Elimination from plasma rate	Bradley et al., 1982	–
*E_*u*_*	1- *E_*b*_*	h^−1^	Plasma's CTC fraction eliminated via urine		–
*E_*b*_*	Uniform (0.39, 0.64)	h^−1^	Plasma's CTC fraction eliminated via bile	Eisner and Wulf, 1963	0.3901, 0.5106, 0.6399
η	Uniform (0.69, 0.89)	–	Fraction of CTC adsorbed to digesta in the small and large intestine		0.69, 0.7893, 0.8899
*bw*	300 (Constant)	kg	Steer body weight		–
*V_*li*_*	Uniform (6, 22)	L	Large intestine contents volume	Volkova et al., 2017	6.0004, 13.9525, 21.9999
*V_*p*_*	0.057^*^bw	L	Volume of plasma	Hansard et al., 1953	–

*For each parameter, the range implemented in the model, parameter units, definition, and references for the parameter value estimates used are listed. The realized parameter range indicates the minimum, median, and maximum (respectively) parameter values from 4,000 simulations (1,000 simulations for each of three treatment scenarios plus the no-treatment scenario)*.

#### *Escherichia coli* population model

Parameter distributions for the *E. coli* population dynamics parameters and parameters of CTC pharmacodynamics against *E. coli* are given in Table 2B. The net growth rate of *E. coli*, accounting for the anaerobic conditions in the cattle large intestine, was adopted from a previously published model (Volkova et al., 2012), in which the growth rate was estimated based on the data of laboratory experiments of *E. coli* growth in animal cecal contents (Freter et al., 1983b). Other literature (Durso et al., 2004) supports that the population growth rate of generic *E. coli* in anaerobic conditions is reduced compared to aerobic growth. We were able to identify only two estimates for the fitness cost of tetracycline resistance in *E. coli* (Nguyen et al., 1989; Ahmad et al., 2015b). In the first study, *E. coli* demonstrating phenotypic tetracycline resistance grew at a rate equal to susceptible *E. coli* in the absence of antimicrobial exposure, indicating no fitness cost of tetracycline resistance (Ahmad et al., 2015b). However, the second study found a fitness cost to *E. coli* from carrying plasmids, excluding the cost of tetracycline genes (1–2% decreased growth rate). Constitutive tetracycline-resistant mutants carry a relatively high fitness cost (2–4%), although inducible tetracycline resistance genes only confer a fitness cost of at most 0.3% (Nguyen et al., 1989). The data for *E. coli* agree with data for *Salmonella*; tetracycline resistance plasmids confer a small fitness cost to *Salmonella* (Gullberg et al., 2011). We assumed that susceptible *E. coli* in our model did not carry conjugative resistance elements and that intermediate and resistant *E. coli* carried plasmids or transposons with inducible tetracycline genes and therefore may experience a fitness cost in the absence of tetracycline. In the absence of additional evidence, we assumed that intermediate and resistant *E. coli* experienced the same fitness cost, which was estimated to have a Uniform distribution from 0 (Ahmad et al., 2015b) to a 3% reduction in the bacterial hourly growth rate (Nguyen et al., 1989; Gullberg et al., 2011).

**Table 2b:** *Escherichia coli* population and pharmacodynamic model parameters.

**Parameter**		**Distribution**	**Unit**	**Definition**	**References**	**Realized Parameter Range**
α_*j*_	α_*s*_	0	–	Fitness cost for susceptible *E. coli*	Nguyen et al., 1989	3.7393e^−6^, 0.0152, 0.03
	α_*i*_	Uniform (0, 0.03)	–	Fitness cost for intermediate *E. coli*		
	α_*r*_	Uniform (0, 0.03)	–	Fitness cost for resistant *E. coli*		
*r*		Uniform (0.05, 0.5)	h^−1^	Specific (maximum) *E. coli* population growth rate	Freter et al., 1983a	0.0501, 0.2759, 0.4999
λ_*in*_		Uniform (0.001, 0.01)	h^−1^	Fractional inflow *E. coli*	Daniels et al., 2009	0.001, 0.0055, 0.01
λ_*out*_		Uniform (0.01, 0.02)	h^−1^	Fractional outflow *E. coli*	Daniels et al., 2009	0.01, 0.015, 0.02
*N_*max*_*		10^(*Weibull*(14.03, 20.32)−7.59)^	*CFU/g*	Carrying capacity for *E. coli* per *g* digesta in the large intestine	Callaway et al., 2002; Krause et al., 2003; Laven et al., 2003; Aslam et al., 2004; Branham, 2007; Lowrance et al., 2007; Alexander et al., 2009, 2010; Agga et al., 2016	10^3.0056^, 10^6.1974^, 10^7.8763^
β_*jj*_		10^(*Gamma*(94.17, 0.16)−22.57)^	h^−1^	Plasmid/transposon transfer rate. The rate for each pair of the *E. coli* subpopulations was drawn from this distribution (resistant-intermediate; resistant-susceptible; intermediate-susceptible).	Levin et al., 1979; Freter et al., 1983b; Simonsen et al., 1990; Gordon, 1992	β_*si*_: 3.651e^−13^, 2.668e^−8^, 0.032 β_*sr*_: 2.339e^−13^, 3.291e^−8^, 0.0514 β_*ir*_: 9.821e^−13^, 3.238e^−8^, 0.0112
*p_*j*_*	*p_*s*_*	1−*p*_*r*_−*p*_*s*_	–	Fraction of inflow *E. coli* that are susceptible	Wagner et al., 2002; Branham, 2007; Lowrance et al., 2007; Alexander et al., 2008; Carson et al., 2008; Platt et al., 2008; Rao et al., 2010; Benedict, 2011; Morley et al., 2011; Kanwar et al., 2013; McGowan, 2014	*p_*s*_*: 0.2439, 0.5311, 0.8188
	*p_*i*_*	Uniform (0.02, 0.15)	–	Fraction of inflow *E. coli* that are intermediate		*p_*i*_*: 0.02, 0.0847, 0.15
	*p_*r*_*	Uniform (0.16, 0.61)	–	Fraction of inflow *E. coli* that are resistant		*p_*r*_*: 0.1601, 0.3877, 0.6099
*start_*j*_*		Same as *p_*j*_*	–	Starting fraction of *E. coli* that are resistant, intermediate, or susceptible		*start_*s*_*: 0.2484, 0.5298, 0.81 *start_*i*_*: 0.0201, 0.0838, 0.15 *start_*r*_*: 0.1601, 0.3846, 0.6098
*E_*max*_*		1 (Constant)	–	$\frac{\text{Growth rate inhibition at}\;CTC_{li\text{\_}conc}=\infty }{\text{Maximum}\;E.coli\text{growth rate}}$	Ahmad et al., 2015a,b	–
*E_0_*		1 (Constant)	–	$\frac{E.coli\text{growth rate at}\;CTC_{li\text{\_}conc}\text{=0}}{\text{Maximum}\;E.coli\text{growth rate}}$	Norcia et al., 1999	–
*H_*j*_*	*H_*s*_*	Uniform (1.62, 2.23)	–	Hill coefficient of susceptible *E. coli*	Ahmad et al., 2015b	*H_*s*_*: 1.6201, 1.9299, 2.23
	*H_*i*_*	Uniform (5.71, 9.53)	–	Hill coefficient of intermediate *E. coli*		*H_*i*_*: 5.7103, 7.6049, 9.5283
	*H_*r*_*	Uniform (6.42, 10)	–	Hill coefficient of resistant *E. coli*		*H_*r*_*: 6.4211, 8.2041, 9.9996
*MIC_*j*_*	*MIC_*s*_*	Uniform (0,4)	μg/mL	Anaerobic minimum inhibitory coefficient of susceptible *E. coli*	Clinical and Laboratory Standards Institute, 2015; DeMars et al., 2016	*MIC_*s*_*: 0.0013, 2.0308, 3.9959
	*MIC_*i*_*	Uniform (2.7,16)	μg/mL	Anaerobic minimum inhibitory coefficient of intermediate *E. coli*		*MIC_*i*_*: 2.7001, 9.6878, 15.9961
	*MIC_*r*_*	Uniform (14.7,128)	μg/mL	Anaerobic minimum inhibitory coefficient of resistant *E. coli*		*MIC_*r*_*: 14.7156, 70.1554, 127.9662

*For each parameter, the range implemented in the model, parameter units, definition, and references for the parameter value estimates used are listed. The realized parameter range indicates the minimum, median, and maximum (respectively) parameter values from 4,000 simulations (1,000 simulations for each of three treatment scenarios plus the no-treatment scenario)*.

In the implemented model, *E. coli* in the enteric population can grow until they reach the carrying capacity; thus, the population growth plus inflowing bacteria equals death plus outflowing bacteria. Estimates of the *E. coli* carrying capacity in the large intestine of feedlot cattle (log_10_CFU/g feces) were obtained from nine studies (*n* = 44 reported averages from 3,610 fecal or colon samples) (Callaway et al., 2002; Krause et al., 2003; Laven et al., 2003; Aslam et al., 2004; Branham, 2007; Lowrance et al., 2007; Alexander et al., 2009, 2010; Agga et al., 2016). Candidate distributions were fit to this data and a Weibull distribution was found to have the best fit (Kolmogorov-Smirnov *P* = 0.125, Anderson-Darling *P* = 0.041), and thus was used to model the inter-individual variability in the large intestine *E. coli* carrying capacity. The total *E. coli* population at time 0 was allowed to vary from 10 to 90% of the carrying capacity.

Plasmids or transposons carrying antimicrobial resistance genes can transfer between susceptible, intermediate, and resistant bacteria, affecting the fraction of *E. coli* within each subpopulation in the large intestine. The rate of conjugative transfer of tetracycline resistance among free-living *E. coli* in the bovine large intestine is not known. *In vitro* density-dependent plasmid transfer rates (mL/cell^*^hour) for *E. coli* and the plasmid R1 were extracted from four studies (*n* = 138 transfer experiments) (Levin et al., 1979; Freter et al., 1983b; Simonsen et al., 1990; Gordon, 1992) and converted to frequency-dependent rates (hour^−1^) by multiplying the density-dependent rate by the initial cell density. The best-fit distribution for the frequency-dependent plasmid transfer rate was a Gamma distribution (Kolmogorov-Smirnov *P* > 0.50, Anderson-Darling *P* = 0.176). In the absence of conjugative element transfer data specifically for tetracycline resistance in *E. coli*, we assumed that the rate of gene transfer from resistant or intermediate bacteria to recipients (susceptible or intermediate bacteria) would come from the plasmid-transfer population parameter distribution described above.

Ingestion of *E. coli* from the pen environment, feed, and water, as well as transit of resident small intestine populations, and shedding of mucosal-associated *E. coli* into the lumen provides an inflow of new bacteria into the large intestine digesta. The defecation of feces containing *E. coli* constitutes the outflow of *E. coli* from the large intestine (Ayscue et al., 2009). We kept the same range of inflow and outflow rates as our previous *E. coli* population model (Volkova et al., 2012) and divided the range between inflow and outflow via an iterative process in order to keep *E. coli* populations at or below carrying capacity and prevent the accumulated losses of *E. coli* (outflow and death) from being greater than accumulated gains (inflow and growth) over the time period of the model. This maintained a stable total *E. coli* population in the model.

We assumed that the majority of ingested *E. coli* came from fecal contamination of the pen environment, feed, and water. We also assumed that the resident small intestinal and mucosal-associated *E. coli* would have the same resistance levels as colon luminal *E. coli*. Therefore, the proportions of inflow bacteria that are resistant or intermediate were estimated from studies of *E. coli* antimicrobial resistance in feedlot cattle feces. These studies cover a wide range of management and sampling strategies so the 10th and 90th percentiles of tetracycline resistance prevalence across these studies were used as the minimum and maximum in a Uniform distribution (Wagner et al., 2002; Branham, 2007; Lowrance et al., 2007; Alexander et al., 2008; Carson et al., 2008; Platt et al., 2008; Rao et al., 2010; Benedict, 2011; Morley et al., 2011; Kanwar et al., 2013; McGowan, 2014) of the resistance in inflow bacteria. Non-overlapping Uniform distributions were used in order to keep the sum of the incoming resistant, intermediate and susceptible *E. coli* proportions at 1. The starting proportions of resistant, intermediate, and susceptible *E. coli* in the large intestine were drawn from the same Uniform distributions.

#### Pharmacodynamic model

Data on the tetracycline *EC*_*50*_ of *E. coli* isolates from cattle is not available, so data for 50 *E. coli* swine isolates were used to determine the relationship between *EC*_*50*_ and MIC (Ahmad et al., 2015a). A linear regression model (deemed appropriate based on the data for swine *E. coli*; Ahmad et al., 2015b) for the value of log_2_(*EC*_*50*_) depending on the isolate log_2_(MIC) was fit in PROC REG in SAS. The resulting model (*R*^2^ = 0.99) is given in Equation 16 (Table 1B) and was used to predict the *EC*_*50*_ of cattle *E. coli* isolates from their MIC. The breakpoints (established by the Clinical and Laboratory Standards Institute) for *E. coli* interpretation in a veterinary infection as resistant, intermediate, and susceptible to tetracycline were used to define the MIC of each of the *E. coli* subpopulations; Uniform MIC distributions were used in the model. The upper limit of resistant MIC considered was 128 μg/mL. The anaerobic MIC penalty was assumed to range between the 1st percentile (−1.3) and 99th percentile (0) reported difference between anaerobic and aerobic tetracycline MIC for generic *E. coli* (DeMars et al., 2016). Therefore the lower-bound of the aerobic MIC distributions was decreased by 1.3, except for the susceptible MIC which could not be less than 0.

The Hill coefficients used in the pharmacodynamic model were based on the estimates for the same set of 50 *E. coli* swine isolates (Ahmad et al., 2015b). A best-fit model for the Hill coefficients could not be found so the interquartile ranges were used as the boundaries of the Uniform distributions of the Hill coefficients for resistant and susceptible *E. coli*. Only one intermediate isolate was examined (Ahmad et al., 2015b) so a ±25% interval around the Hill coefficient value for this isolate was used as the distribution boundaries for the intermediate *E. coli* Hill coefficient.

### Model implementation

The differential equations of the model were implemented in MatLab® R2016b (MathWorks, Natick, MA, U.S.) using a time-step of 0.1 h. The *Escherichia coli* population was allowed to reach equilibrium of the resistant, intermediate, and susceptible proportions before CTC was fed. The model was run for a 90 day simulation period, including the initial 48 h required to reach the pre-treatment equilibrium (Day 0–2), the treatment period (Day 2–10 for ADT, Day 2–30 for ARLA and ADC), and post-treatment period (Day 11–90 for ADT, Day 31–90 for ARLA and ADC). A thousand simulations were run for each treatment scenario, representing 1,000 treated cattle; simulations were also run for a 1,000 cattle with no CTC treatment. Descriptive statistics of the *E. coli* subpopulations were calculated in MatLab using the IOSR statistics toolbox. The bacterial subpopulation proportion medians were tested for equality with Kruskal-Wallis tests followed by Dunn-Sidak *post-hoc* comparisons. All significance tests were two-sided with α = 0.05. The following time-periods were defined for further investigation: *pre-treatment* (hour 42–48), *during treatment* (the steady-state from hour 552 to 720 for ARLA and ADC; hour 48 to 240 for ADT), *day 90* (last 24 h), *0-day withdrawal period* (6 h after treatment ends), *1-day withdrawal period* (24 h after treatment ends), and *2-day withdrawal period* (48 h after treatment ends). Figures were created in MatLab using the IOSR statistics toolbox and SubPlot toolbox. The MatLab code for the model is provided in Supplementary Materials.

## Results

All three scenarios of CTC indications for beef cattle (antimicrobial liver abscess reduction—ARLA; disease control—ADC; disease treatment—ADT) were implemented in the model of CTC pharmacokinetics-pharmacodynamics and generic *E. coli* population dynamics in the bovine large intestine. The average CTC concentration in the large intestine during treatment was calculated for the median steer, 5th percentile steer, and 95th percentile steer. For ARLA, the median steer had an average large intestine CTC concentration of 0.22 μg/mL, the 5th percentile steer had a concentration of 0.06 μg/mL, and the 95th percentile steer had a concentration of 0.62 μg/mL. For steers given ADC, the averages were 1.17, 0.34, and 2.96 μg/mL for median, 5th percentile and 95th percentile, and for ADT the average CTC concentrations were 12.99, 3.70, and 34.12 μg/mL, respectively. This is consistent with the results from our previous deterministic pharmacokinetic model (Cazer et al., 2014).

**Figure 1:**
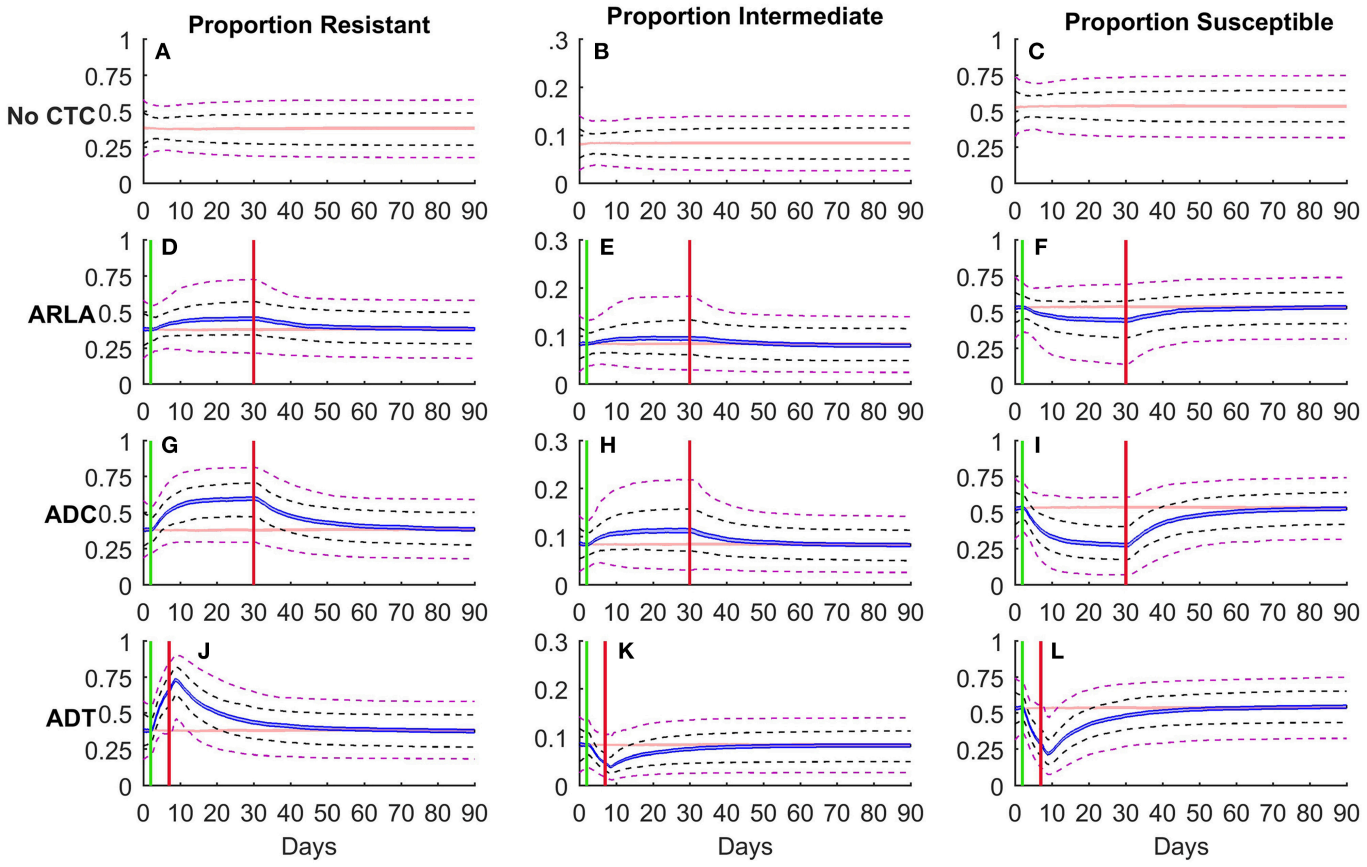
Simulation of *Escherichia coli* subpopulations (resistant [**A, D, G, J**]; intermediate [**B, E, H, K**]; susceptible [**C, F, I, L**]) in the large intestine of beef cattle in the presence **(D–L)** and absence **(A–C)** of oral chlortetracycline treatment. Proportions (Y-axis) are presented for 1,000 simulations of each treatment scenario. Shaded band is the 95% non-parametric confidence interval of the median, black dashed lines are 25 and 75% percentiles and purple dashed lines are 5 and 95% percentiles of the overall distribution. The red shaded band represents the median proportion in the absence of chlortetracycline (CTC) treatment. Blue shaded bands represent the median proportion in antimicrobial treatment scenarios for reduction of liver abscesses (ARLA; **D–F**), disease control (ADC; **G–I**) or disease treatment (ADT; **J–L**). The green and red vertical lines mark the beginning and end of CTC treatment, respectively.

The use of CTC for any indication resulted in an increase in the median proportion (Figures 1D,G,J) and absolute number (Figure S1) of resistant *E. coli* during treatment in 1,000 simulated cattle. The median proportion resistant was 38% before treatment and increased to 46% at the end of ARLA, 60% at the end of ADC, and 66% at the end of ADT. In terms of the number of resistant *E. coli* in the large intestine, there were 10^5.76^ CFU/g before treatment, 10^5.82^ CFU/g at the end of ARLA, 10^5.93^ CFU/g at the end of ADC, and 10^5.89^ CFU/g at the end of ADT. The median proportion of intermediate *E. coli* also increased during ARLA (10% at the end of treatment) and ADC (11%) but decreased during ADT (5%) relative to pre-treatment levels (8%) (Figures 1B,E,H,K). However ADT still greatly increased the median proportion (71% at the end of ADT) and amount (10^5.92^ CFU/g) (Figure S1) of non-susceptible bacteria compared to before treatment (46%; 10^5.84^ CFU/g), accompanying the drop in the median proportion of susceptible *E. coli* (54% before treatment and 29% at the end of ADT) (Figure 1L). The proportions and amounts of resistant, intermediate, and susceptible bacteria reached an approximate steady state during ARLA and ADC (Figures 1D,G; Figure S1), whereas a steady state was not reached during the shorter ADT and the proportion of resistance peaked, on average, on the second day after ADT treatment was discontinued (Figure 1J). In general, ARLA and ADC resulted in an upward shift of the median and upper limit (95%) of the proportion non-susceptible distribution but little change in the lower limit (5%) during antimicrobial treatment (Figures 1F,I). In contrast, during the ADT period and following 3 days the entire proportion resistant distribution shifted upwards (Figure 1J).

The median proportion of resistant *E. coli* reversed to the pre-treatment median confidence interval 15 days after ARLA ended, 31 days after ADC ended, and 36 days after ADT ended (Figures 1B–D). Only 35% of ARLA cattle, 50% of ADC cattle and 61% of ADT cattle returned to within 10% of their individual pre-treatment resistant *E. coli* proportion by the end of the 90 day simulation period. This includes 18% of ARLA cattle, 9% of ADC cattle, and 6% of ADT cattle that were already within 10% of pre-treatment levels at the end of antimicrobial treatment. Among the individual cattle that did return to pre-treatment levels, the median time from the end of treatment to the resistance reversion was significantly different between the treatment groups (*P* < 0.001): <1 day (mean 6 days) following ARLA, 8 days (mean 11 days) following ADC, and 11 days (mean 15 days) following ADT. Since ADT duration is shorter, the 90 day simulation period captured 60 days post-ARLA and -ADC but it captured 83 days post-ADT. At 60 days post-treatment, 59% of ADT cattle had returned to within 10% of pre-treatment proportion resistant with a median return time of 11 days (mean 14 days) after the treatment.

**Figure 2:**
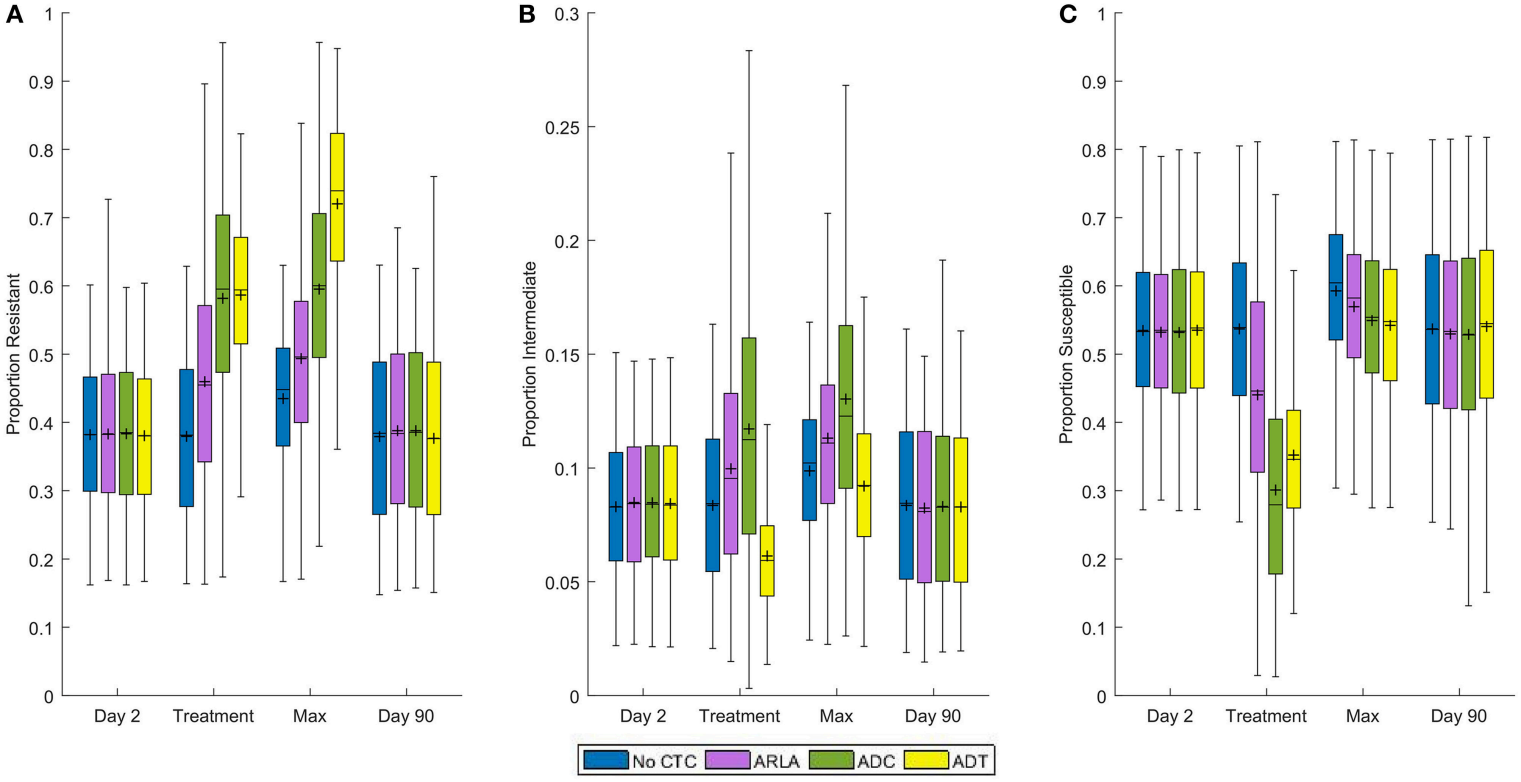
Distribution of proportions of tetracycline resistant **(A)**, intermediate **(B)**, and susceptible **(C)** *Escherichia coli* in the large intestine of beef cattle at four time periods. *Day 2* is the mean proportion during the 6 h before chlortetracycline treatment starts. *Treatment* is the mean proportion from Day 2 to 10 for ADT and from Day 23 to 30 for ARLA, ADC, and in the absence of treatment. *Max* is the maximum proportion between Days 2 and 12 for ADT and between Days 2 and 35 for ARLA, ADC, and in the absence of treatment. *Day 90* is the mean proportion during the 24 h of the last day of the simulation period. Shaded box extends from 25^th^ to 75^th^ percentile of the simulated proportion. Middle line is the median and plus-symbol is the mean. Whiskers extend to the minimum or maximum data point within 1.5 times the interquartile range from the first or third quartile, respectively. The results of 1,000 simulations of each treatment scenario are summarized in each boxplot.

In the absence of treatment, 38% of generic *E. coli* in the cattle large intestine were resistant, 8% intermediate, and 54% susceptible to CTC on average (Figure 2). There was no significant difference in the average proportions of resistant bacteria during ADC (58%) and ADT (59%) (*P* = 0.850, Figure 2A), but ADC had a significantly higher proportion of intermediate bacteria (12%) compared to ADT (6%) (*P* < 0.001) (Figure 2B). This was associated with ADC having a lower average susceptible proportion of *E. coli* (30%) than ADT (35%, Figure 2C) despite ADT having the highest maximum proportion resistant (72%). At the steady state during ARLA, 46% of *E. coli* were resistant, 10% intermediate, and 44% susceptible on average. The average proportion of resistant *E. coli* during any of the three CTC treatments was significantly different from that in the absence of treatment (38%) (*P* < 0.001, Figure 2A). By the end of the 90 day simulation period, the mean proportion resistant reversed and was not significantly different from that in the absence of treatment (*P* > 0.050, Figure 2A).

**Figure 3:**
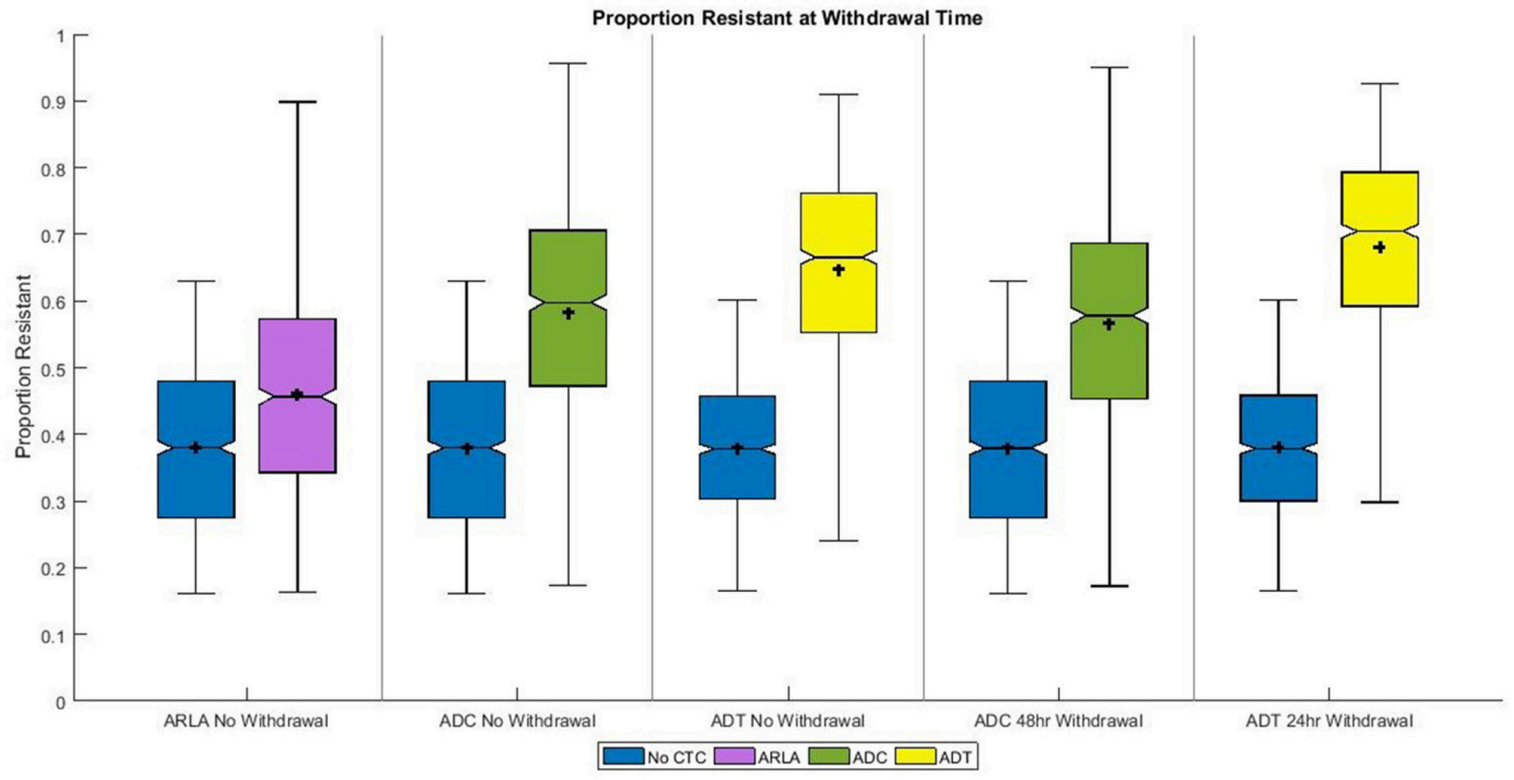
Distribution of proportion tetracycline resistant *Escherichia coli* in the large intestine of beef cattle at the current withdrawal times. For “0 Day” withdrawal period (e.g., “No Withdrawal”) the proportion resistant averged over the 6 h after treatment ended is given. For “48 h Withdrawal” and “24 h Withdrawal” periods, the proportion resistant averaged over 48 and 24 h, respectively, after treatment ended is given. Shaded box extends from 25^th^ to 75^th^ percentile of the simulated proportion resistant. Middle line is the median and plus-symbol is the mean. The notch is the 95% non-parametric confidence interval of the median. Whiskers extend to the minimum or maximum data point within 1.5 times the interquartile range from the first or third quartile, respectively. The results of 1,000 simulations of each treatment scenario are summarized in each boxplot.

Brand-name U.S. CTC feed products have a 0-day meat withdrawal period for beef cattle so they can be sent to slaughter immediately after the consumption of CTC. We assumed that 6 h elapse between leaving the feedlot and slaughter. The median cattle given ARLA had 46% resistant enteric *E. coli* at slaughter; the median ADC cattle had 60% resistant *E. coli*, and the median ADT cattle had 67% resistant *E. coli* (Figure 3). Generic U.S. CTC products have a 2-day withdrawal period following ADC and a 1-day withdrawal period following ADT. These withdrawals resulted in statistically significant different proportions of resistant *E. coli* at slaughter compared to the 0-day withdrawal: the median cattle had 58% resistant *E. coli* at 2 days after ADC (*P* = 0.012) and 71% resistant 1 day after ADT (*P* < 0.001) (Figure 3). The cattle treated with CTC had a wider distribution and greater maximum proportion resistant at the currently recommended withdrawal times compared to those in the absence of treatment (Figure 3). The lower bound of the resistance distribution at the withdrawal times was higher for ADT relative to the other two treatments (Figure 3).

**Figure 4:**
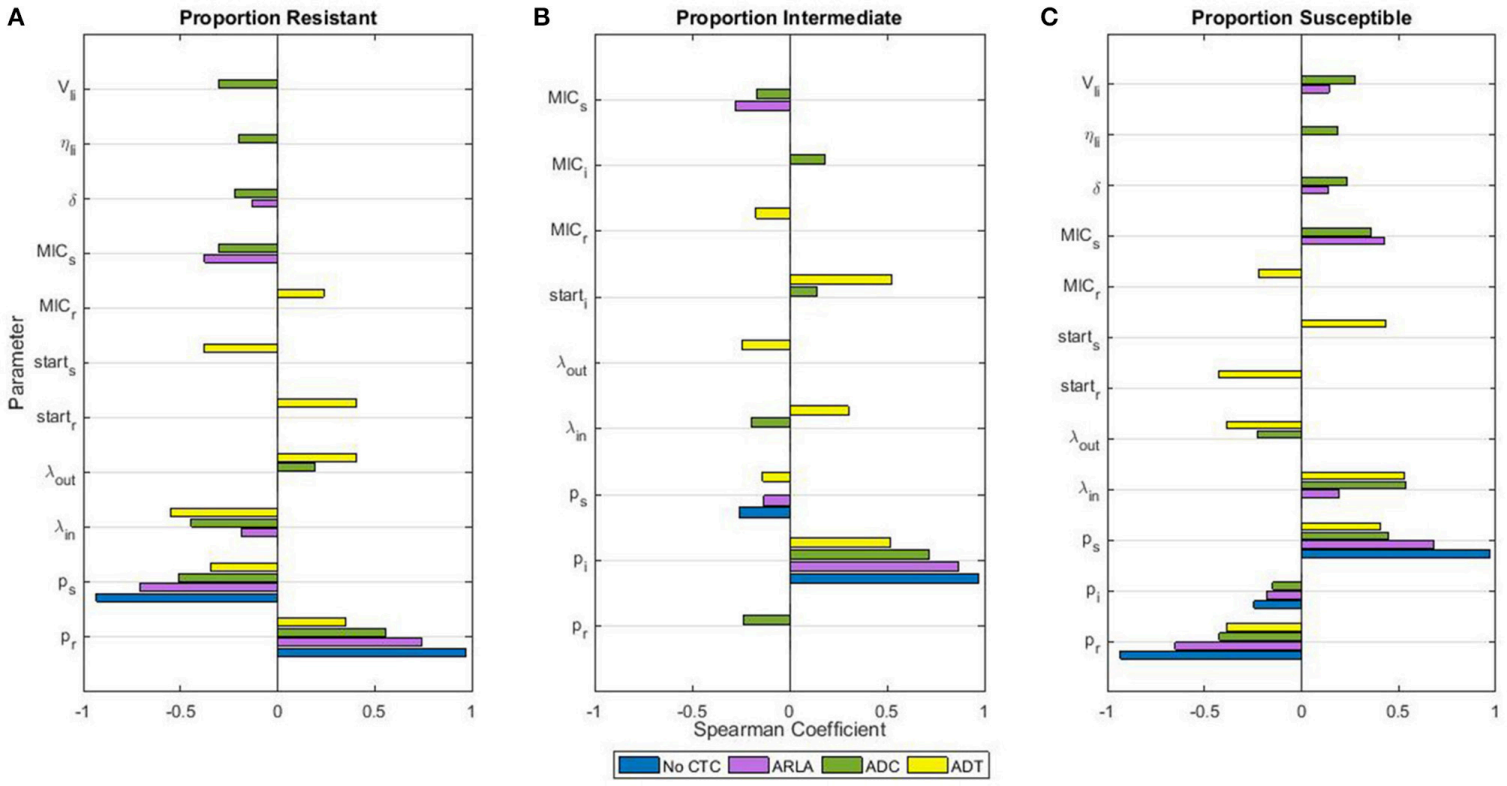
Sensitivity of proportions of tetracycline resistant **(A)**, intermediate **(B)**, and susceptible **(C)** *Escherichia coli* in the large intestine of beef cattle during chlortetracycline treatment to the parameter values of the drug pharmacokinetics, pharmacodynamics, and the bacterial population dynamics. Only parameters that significantly correlate with the proportion (Spearman correlation coefficient *P* < 0.0006, Bonferroni correction) are included. Parameters are defined in Table 2. The time period “during chlortetracycline treatment” was from Day 2 to 10 for ADT and from Day 23 to 30 for ARLA, ADC, and in the absence of treatment. The correlations between the mean proportions during those time periods and parameter values were evaluated using the outputs of 1,000 simulations of each treatment scenario.

Before and after CTC treatments, the proportions of CTC resistant, intermediate, and susceptible enteric *E. coli* in the large intestine were sensitive to variability in the inflowing *E. coli* distribution of resistance (*p*_*j*_). During the treatments, the proportions were sensitive to variability in the bacterial population dynamics parameters and in the CTC pharmacokinetic-pharmacodynamic parameters (Figure 4, Spearman coefficients smaller than the Bonferroni corrected α = 0.0006 are shown). In general, higher CTC dosages made the proportions less sensitive to the distribution of resistance in the inflowing *E. coli* but more sensitive to the overall inflow rate (λ_*in*_) (Figure 4). In addition to the inflow rate and the inflow resistance distribution, the proportions during ADT were sensitive to the starting proportions (*start*_*j*_) and the CTC minimum inhibitory concentration (MIC) for the resistant *E. coli*. Conversely, the proportions during ARLA and ADC were sensitive to the MIC for the susceptible *E. coli*. Variability in the following pharmacokinetic parameters had a significant impact on the *E. coli* proportions only during ARLA and ADC (Figures 4A,C): CTC abiotic degradation throughout the gastrointestinal tract and other organs (δ), CTC binding to the digesta in the large intestine (η), and volume of the digesta in the large intestine (*V*_*li*_). Variability in the Hill coefficient (*H*_*j*_), a pharmacodynamic parameter, had no significant impact on the *E. coli* proportions during treatments. Variability in the bacterial population dynamics parameters of the growth rate (*r*), resistance fitness cost (α), carrying capacity (*N*_*max*_), and the resistance horizontal transfer rate (β), plus the pharmacokinetic parameters of the CTC flow rates through the gastrointestinal compartments (γ_*s*_, γ_*upper*_*si*_, γ_*rest*_*si*_, γ_*li*_) and the fraction excreted in bile (*E*_*b*_), had no significant effect on the proportions of resistant, intermediate, and susceptible *E. coli* during treatments.

## Discussion

Despite modeling a long follow-up period after ending CTC treatment (60 days after ARLA and ADC, 83 days after ADT), a significant number of the simulated cattle had still not returned to their pre-treatment proportion of resistant *E. coli* after CTC treatment. The dosage of CTC appeared to be associated with the percent of cattle that returned to their pre-treatment levels, with higher dosages (ADT, ADC) having greater proportions of cattle returning to pre-treatment resistance than low-dosage ARLA. This suggests that the low-dosage long-term ARLA may more frequently shift the enteric *E. coli* equilibrium to a state that is stable in the absence of CTC. This is evident in the change in proportion resistant from the end of treatment to the end of the simulation period (day 90). ARLA cattle that never returned to pre-treatment levels (65% of ARLA cattle) had a median difference in proportion resistant of 1.7% from the end of ARLA to day 90, indicating that they had reached an equilibrium during treatment that was relatively stable in the absence of CTC. On the other hand, ARLA cattle that did return to pre-treatment levels (35% of ARLA cattle) had a larger median change in proportion resistant (6.4%) from the end of treatment to the end of the simulation period. It is possible that the high levels of resistance observed during ADT and ADC were unsustainable after treatment given the model parameters (particularly the resistant inflow proportion). Only 2.4% of ADC cattle and 4.9% of ADT cattle achieved an equilibrium during treatment that was relatively stable in the absence of CTC (<1.7% difference between end of treatment and day 90 proportion resistant). However, the ARLA cattle that returned to their pre-treatment levels did so much faster than ADC and ADT cattle, likely because there was a smaller change in resistance level from pre-treatment to the end of ARLA (Figure 1).

Additional investigation is needed to study the impact of treatment duration separately from dosage, including testing off-label ADT treatment durations. Pulse-therapy with ADT dosages (3 treatments, 1 day apart) has been implemented in a beef cattle field study; an approximate steady state of 80% resistant *E. coli* isolates was reached by the second treatment (double the pre-treatment proportion of 40%) (Platt et al., 2008). This is similar to the mean pre-treatment proportion resistant (38%) and the mean maximum proportion resistant after ADT (72%) in our study. At the end of the 17 day post-treatment follow-up period, the proportion of resistant isolates in that study was still 1.5 times greater than the pre-treatment proportion. If the decrease continued at the same rate, the proportion resistant would reverse to the pre-treatment level 34 days after the treatment ended, similar to the 36 days required in our model for the median proportion resistant to be within 10% of the pre-treatment median. Additionally, the average proportion resistant during ADT in our study (59%) fell within the 95% confidence interval of the average proportion resistant during ADT pulse-therapy (58.7–77.3%) (Platt et al., 2008), confirming that our model is projecting biologically plausible resistance dynamics.

Unfortunately it is difficult to compare our model results to the data from field trials on *E. coli* tetracycline resistance prevalence following CTC treatment due to the variable field study design and management conditions. Those include pooling of isolates across treatment groups and time (Alexander et al., 2008), combination antimicrobial therapy (Kanwar et al., 2013), and reporting of animal-level rather than isolate-level results (Sharma et al., 2008). In order to fully validate the resistance prevalence predictions of this model, field studies are required that sample individual animals, determine the prevalence of tetracycline resistant *E. coli* isolates before and at several points after CTC treatment, and do not alter diet or environmental conditions during the study period.

This model illustrated important differences in individual and population level antimicrobial resistance. Each simulated population of 1,000 cattle reached its pre-treatment population distribution of tetracycline resistance within the follow-up period (Figure 1). This return occurred approximately 2 weeks after ARLA and 4–5 weeks after ADC or ADT ended. Even though the resistance levels of many individual animals were still significantly altered by CTC treatment, the population or herd resistance was able to return to its initial distribution. This implies that there would be no net increase in resistance exported from the feedlot to slaughterhouses if a sufficient antimicrobial resistance withdrawal period was applied.

The resistance dynamics following different dosages should be considered in antimicrobial use policies. When Denmark stopped using growth promoting doses of antimicrobials (like ARLA) in weaner pigs, the amount of antimicrobials used for disease treatment (like ADT) increased due to higher disease incidence (Kjeldsen and Callesen, 2006). A similar change in the use of CTC may result in significant increases in resistance exported from farms to slaughterhouses, depending on disease incidence and CTC withdrawal period before slaughter. However, long-term ARLA or ADC may release significantly more resistant *E. coli*, cumulatively, into the feedlot environment compared to one 5-day course of ADT. This highlights the need for additional rigorous, longitudinal trials comparing CTC labeled uses and collecting data on *E. coli* in cattle, in the feedlot environment, and on beef products.

Based on the model outcomes, it is evident that the current U.S. withdrawal periods (designed to mitigate violative drug residues in the edible meat) for in-feed CTC (Zoetis Aureomycin 50; ChlorMax 50; Hoffmann La-Roche Inc., 1997) in beef cattle are insufficient to reduce the treatment-associated prevalence of resistant *E. coli* in the large intestine, which can contaminate processing plants and beef products at slaughter (Sheridan, 1998; Aslam et al., 2003). Currently, U.S. cattle can consume CTC until immediately prior to slaughter and hence the proportion of tetracycline resistant bacteria in their intestine is significantly greater than in untreated cattle (Figure 3). The 24-h withdrawal after ADT may further increase the proportion of resistant *E. coli* at slaughter because of the CTC intestinal transit times.

Apart from the United States, the only other country with a large beef cattle industry that permits the use of oral CTC is Canada. They allow CTC fed at 70 mg/head per day (ARLA dosage) to prevent foot rot in beef cattle with a pre-slaughter withdrawal period of 5 days (Canadian Food Inspection Agency, 2016). Canada also has an approved CTC-sulfamethazine combination product that is fed at 350 mg CTC per head per day (ADC dosage) with a 10 day pre-slaughter withdrawal period (Canadian Food Inspection Agency, 2016). The European Union, United Kingdom, Australia, New Zealand, Brazil, Argentina, and Mexico have no approved oral CTC products for beef cattle over 6 months old, although a few permit topical CTC sprays with a 0 day pre-slaughter withdrawal period and New Zealand has an approved intrauterine CTC product with a 7 day pre-slaughter withdrawal period (P.R. Vademecum^1^; Australian Pesticides and Veterinary Medicines Authority, 2015; EU Directorate General Health Consumers, 2017; New Zealand Ministry for Primary Industries, 2017; UK Veterinary Medicines Directorate, 2017). This modeling study demonstrated that a 15 day withdrawal after ARLA, a 31 day withdrawal after ADC, and a 36 day withdrawal after ADT (Figure 1) may be necessary to reduce the median prevalence of tetracycline resistance among enteric *E. coli* that could contaminate beef products. Such withdrawal periods would be longer than any current CTC withdrawal periods (which are currently used to control only the CTC residues in the animal edible tissues) in countries with large beef industries. Similar or longer withdrawal periods have been used in antimicrobial feeding studies without detriment to cattle health and welfare (Alexander et al., 2008; Sharma et al., 2008; Agga et al., 2016). However, the economic and welfare costs of resistance withdrawal periods will depend on the specific disease treated and management strategy.

The same field studies (Alexander et al., 2008; Agga et al., 2016) did not find the current withdrawal periods effective in decreasing tetracycline resistance levels. However, one study (Alexander et al., 2008) observed a large increase in tetracycline resistant fecal *E. coli* when switching from a silage to grain based diet and this effect overwhelmed the ability to detect any changes due to antimicrobial withdrawal. The second study (Agga et al., 2016) found a decrease in fecal *E. coli* tetracycline resistance during a 27-day withdrawal after ADT but then observed a steady increase in both control and treated groups for an additional 90 days. They concluded that resistance is selected for in cattle-occupied pens independently of antimicrobial use (Agga et al., 2016). Since our model includes only effects of the administered antimicrobials, it is unable to simulate the pattern they observed. The model could be expanded to include the effects of diet (Alexander et al., 2008; Volkova et al., 2016, 2017), environmental *E. coli* populations (Ayscue et al., 2009; Volkova et al., 2013), environmental contamination with antimicrobial drugs (Call et al., 2013), and animal feeding or environmental contamination with other antimicrobial compounds such as heavy metals (Berendonk et al., 2015).

The sensitivity of the outputs of our model to *E. coli* population dynamics parameters, particularly the inflow and outflow of *E. coli* in the large intestine, emphasizes the lack of knowledge about *E. coli* and antimicrobial resistance circulation in feedlot environments. It also suggests that interventions aimed at changing the turnover of *E. coli* in the large intestine and the incoming proportions of resistant bacteria may be highly effective at reducing antimicrobial resistance. For example, moving cattle to a previously unoccupied pen for the resistance withdrawal period may reduce the ingestion of resistant bacteria (Agga et al., 2016) and shift the enteric population to a lower-resistance equilibrium. Additional research is needed on the circulation of antimicrobial resistant bacteria and genes in the feedlot environment and how the environmental bacterial populations intersect with the enteric ones. Probiotics and some feed additives have been shown to alter *E. coli* O157 dynamics in beef cattle (Sargeant et al., 2007) and similar strategies could complement an antimicrobial withdrawal period. Altering pharmacokinetic parameters, such as CTC degradation rate in the gastrointestinal tract or its sorption to digesta, may also be an avenue for decreasing the antimicrobial exposure of enteric microbiomes.

The level of resistant bacteria during CTC treatment was not significantly associated with the fitness cost value or the plasmid transfer rate value in the global sensitivity analysis. These findings are consistent with mathematical models of tetracycline resistant *E. coli* in the swine intestine, which found that the effect of the plasmid transfer rate was inconsequential compared to the effect of the growth rate of resistant bacteria (Graesboll et al., 2014; Ahmad et al., 2015a). The same models also found that resistant bacteria could persist in the swine intestine with a fitness cost up to an 80% reduction in the growth rate (Graesboll et al., 2014), although this was much greater than the fitness cost they observed *in vitro* (Ahmad et al., 2015b). Consistent with our model of tetracycline resistance in *E. coli* in the cattle intestine, the models of tetracycline resistant *E. coli* in the swine intestine found that bacterial inflow and outflow had a stronger correlation with the level of resistance during treatment than fitness cost or plasmid transfer rates (Graesboll et al., 2014).

By necessity, mathematical models are a simplified representation of biologic systems and can omit confounding or integral parameters. Since our model was limited to modeling *E. coli* within the large intestine, we were unable to directly account for environmental factors that may impact resistance dissemination and persistence in beef feedlots. In addition, this model did not account for co-selection of CTC resistance as a result of administering other antimicrobials, which may be an important mechanism for resistance dissemination and persistence in food animals (Love et al., 2016). The model parameter value distributions were assigned based on the available published literature. However, specific estimates of the relevant parameters for *E. coli* and CTC in cattle are often lacking and the parameter value distributions built on sparse data from cattle and other species introduce an unknown amount of error into the model outputs. In addition, *in vitro* data were used when *in vivo* data were not available for parameterization. This should be considered when interpreting the model outputs. For example, two *in vitro* estimates of tetracycline fitness cost in *E. coli* were used to parameterize fitness cost in our model (Nguyen et al., 1989; Ahmad et al., 2015b), resulting in a narrow range of fitness cost values (Table 2B). We performed additional model simulations considering all biologically possible values of fitness cost by specifying Uniform (0, 0.99) for the fitness cost parameter (data not shown). In that case, the median withdrawal period for ADT decreases from 36 days to 12 days.

In conclusion, our model of CTC pharmacokinetics-pharmacodynamics and the enteric bacterial population dynamics in beef cattle demonstrated that a withdrawal period before slaughter can be effective in reducing the population distribution of tetracycline resistant *E. coli* to pre-treatment levels. However, the withdrawal periods necessary to mitigate this microbiological food safety risk are significantly longer than current CTC withdrawal periods designed to mitigate toxicological food safety risks. The dynamics of resistant *E. coli* during and after CTC treatment vary by CTC dosage.

## Author contributions

CC developed the chlortetracycline pharmacokinetic model, adapted the other model components for this research, parameterized the model, edited the MatLab code, analyzed the model outputs, and drafted this manuscript. LD adapted model components for chlortetracycline pharmacodynamics and tetracycline-resistant *Escherichia coli* in cattle, wrote the MatLab code, and revised this manuscript. VV developed the pharmacodynamic and *Escherichia coli* population models, contributed to the pharmacokinetic model development and model parameterization, and revised this manuscript. YG contributed to the development of the models, model parameterization, model output analysis, and revised this manuscript.

### Conflict of interest statement

The authors declare that the research was conducted in the absence of any commercial or financial relationships that could be construed as a potential conflict of interest.

## Footnotes

^1^P.R. Vademecum P.R. Vademecum: Búsqueda libre de medicamentos, principios activos. [Online]. Available online at: www.prvademecum.com/paises.php (Accessed July 18, 2017).

^2^Zoetis “Aureomycin 50 Granular A [package insert]”, in: Chlortetracycline Type A Medicated Article.). http://www.zoetisus.com/contact/pages/product_information/msds_pi/pi/aureomycin_50.pdf.

^3^Zoetis “ChlorMax 50 [package insert]”, in: Chlortetracycline Type A Medicated Article Antibacterial.). http://www.zoetisus.com/contact/pages/product_information/msds_pi/pi/ChlorMax_50.pdf.

## Abbreviations

CTC: chlortetracycline
ARLA: antimicrobial reduction of liver abscesses
ADC: antimicrobial disease control
ADT: antimicrobial disease treatment
MIC: minimum inhibitory concentration.
