# Corrigendum: Monte Carlo Simulations Suggest Current Chlortetracycline Drug-Residue Based Withdrawal Periods Would Not Control Antimicrobial Resistance Dissemination from Feedlot to Slaughterhouse

**DOI:** 10.3389/fmicb.2018.00949

**Published:** 2018-05-08

**Authors:** Casey L. Cazer, Lucas Ducrot, Victoriya V. Volkova, Yrjö T. Gröhn

**Affiliations:** ^1^Department of Population Medicine and Diagnostic Sciences, College of Veterinary Medicine, Cornell University, Ithaca, NY, United States; ^2^Department of Diagnostic Medicine/Pathobiology, Institute of Computational Comparative Medicine, College of Veterinary Medicine, Kansas State University, Manhattan, KS, United States

**Keywords:** beef cattle, antibiotic resistance, enteric bacteria, food-borne pathogens, mathematical modeling, population pharmacokinetics, pharmacodynamics

In Table 1B, Equations 14a–c, the denominators (e.g., *N*_*s*_ + *N*_*i*_) should be the entire *Escherichia coli* population (*N* = *N*_*s*_ + *N*_*i*_ + *N*_*r*_). The corrected Equations 14a–c appear below.

In the MATLAB code provided in the Supplementary Materials, the denominator of Equation 14 (plasmid_transfert_si, plasmid_transfert_sr, plasmid_transfert_ir) was, correctly, the entire *E. coli* population. This correction does not impact the scientific conclusions of the article in any way. The authors apologize for this mistake.

The original article has been updated.

**Table 1B:** *Escherichia coli* population and pharmacodynamic model equations.

**Equation number**	**Equation**	**Description**
14	a) *PT*_*is*_ = $\beta \frac{N_{s}N_{i}}{N}$ b) *PT*_*rs*_ = $\beta \frac{N_{s}N_{r}}{N}$ c) *PT*_*ri*_ = $\beta \frac{N_{i}N_{r}}{N}$	Transfer of plasmids/transposons from (a) intermediate to susceptible, (b) resistant to susceptible, and (c) resistant to intermediate *E. coli*. β is the rate of plasmid transfer between two *j* populations of *E. coli, N_*j*_* is the number of *j*^1^ *E. coli* in the large intestine, and *N* is the total number of *E. coli* in the large intestine.

1 *j* population refers to *s* (susceptible), *i* (intermediate resistance), or *r* (resistant).

## Conflict of interest statement

The authors declare that the research was conducted in the absence of any commercial or financial relationships that could be construed as a potential conflict of interest.

