# Supplementary material for: Monte Carlo Simulations Suggest Current Chlortetracycline Drug-Residue Based Withdrawal Periods Would Not Control Antimicrobial Resistance Dissemination from Feedlot to Slaughterhouse
Source: Front Microbiol. 2017 Sep 20;8:1753. doi: 10.3389/fmicb.2017.01753 (PMC5627025; doi:10.3389/fmicb.2017.01753)
Supplement: Supplementary file 2 [file DataSheet1.docx]

Supplementary Material

**Monte Carlo Simulations Suggest Current Chlortetracycline Drug-Residue Based Withdrawal Periods Would Not Control Antimicrobial Resistance Dissemination from Feedlot to Slaughterhouse**

**Casey L. Cazer^*^, Lucas Ducrot, Victoriya V. Volkova, Yrjö T. Gröhn**

*** Correspondence:** Casey L Cazer: clc248@cornell.edu

**Supplementary Figures**


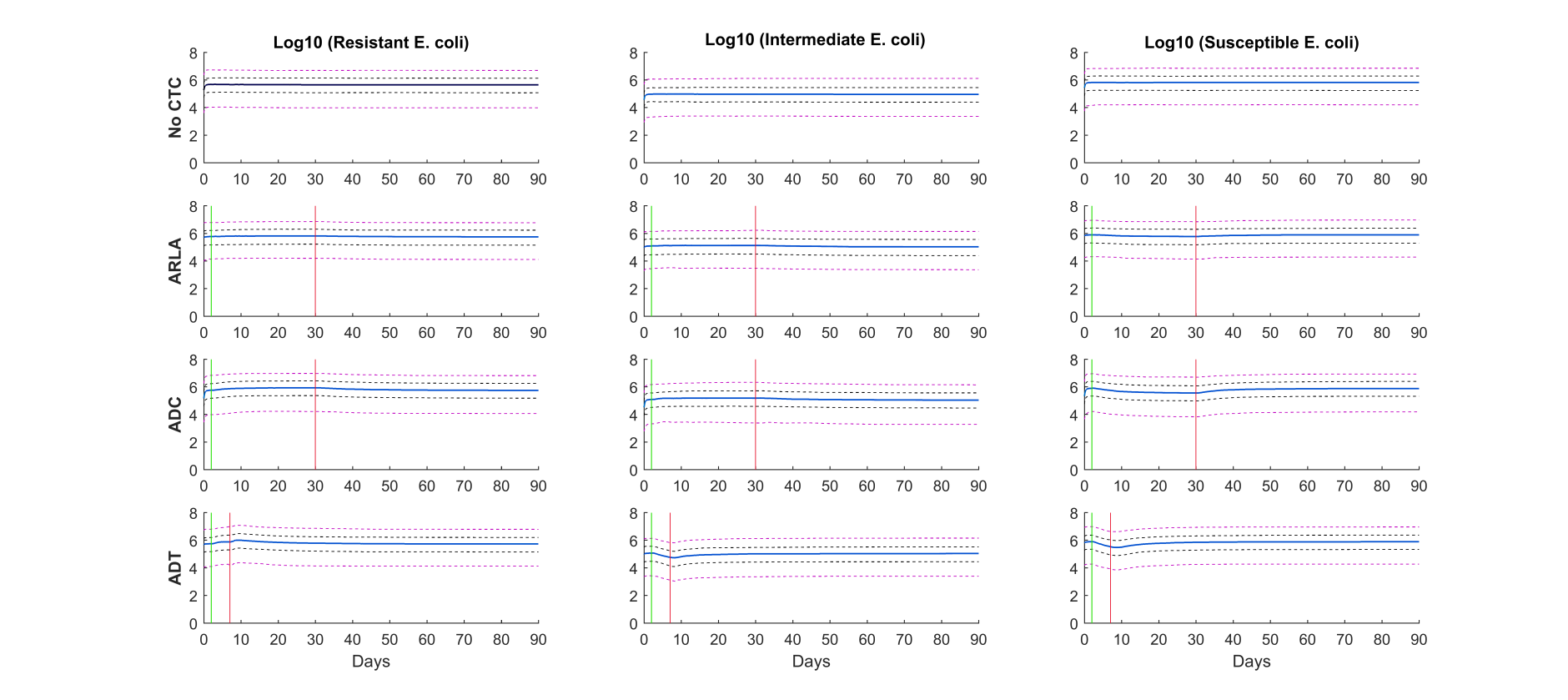


**Figure S1**: Number of resistant, intermediate and susceptible *Escherichia coli* in the bovine large intestine with and without oral chlortetracycline treatment. The log_10_(*E. coli*) (Y-axis) are presented for 1,000 simulations of each treatment scenario (rows): the absence of chlortetracycline (CTC) treatment, CTC for the reduction of liver abscesses (ARLA), disease control (ADC) or disease treatment (ADT). Blue shaded band is the 95% non-parametric confidence interval of the median, black dashed lines are 25% and 75% percentiles and purple dashed lines are 5% and 95% percentiles of the overall distribution. Treatment durations are the same as in Figure 1.

**Supplementary Information**

**MATLAB Model Code**

%{

Abbreviations:

ARLA: antimicrobial reduction of liver abscesses

ADC: antimicrobial disease control

ADT: antimicrobial disease therapy

PD: pharmacodynamic

PK: pharmacokinetic

Pop: population

eco: E coli

li: large intestine

CTC: chlortetracycline

ss: steady state

prop: proportion

res: resistant

int: intermediate-resistant

susc: susceptible

SIR, xx: susceptible/intermediate/resistant

conc: concentration

st: stomach

upsi: upper small intestine

rtsi: rest of small intestine

pl: plasma

tis: tissue

amt: amount

cat: cattle

deg: degradation

tx: treatment

}%

INTRASTOCHA=0; %1 intra-individual stochasticity (square root terms)

%0=no stochastic;

%intra-individual stochasticity was not implemented in this model;

PKSTOCHA=1;

%1 interindividual stochasticity of PK parameters; %0 no stochastic;

POPSTOCHA=1;

%1 interindividual stochasticity of E. coli population parameters; %0 no

%stochastic;

PDSTOCHA=1;

%1 interindividual stochasticity of PD parameters; %0 no stochastic;

METHODE=1;

% 0=no CTC 1=ARLA; 2=ADC 3=ADT;

time=90*24; %90 day simulation period;

dt=0.1;

am_start=48; %when e coli population has established steady state

adt_time=5*24;

adc_time=28*24;

bw=300; %body weight;

%set up number of simulations;

if INTRASTOCHA==0 && PKSTOCHA==0 && POPSTOCHA==0 && PDSTOCHA==0

n=1; %deterministic model

else

n=1000;

end

%making empty arrays to store stochastic simulations;

res_eco_li=zeros(time/dt,n);

int_eco_li=zeros(time/dt,n);

susc_eco_li=zeros(time/dt,n);

total_eco=zeros(time/dt,n);

%number of e coli in large intestine;

total_eco_ss_preCTC=zeros(1,n);

total_eco_ss_CTC=zeros(1,n);

total_eco_ss_postCTC=zeros(1,n);

prop_res_eco=zeros(time/dt,n);

prop_int_eco=zeros(time/dt,n);

prop_susc_eco=zeros(time/dt,n);

%proportion (percentage) of SIR out of total e coli;

prop_susc_ss_postCTC=zeros(1,n);

prop_int_ss_postCTC=zeros(1,n);

prop_res_ss_postCTC=zeros(1,n);

%proportion of SIR out of total e coli at end of simulation;

t480_susc_eco_li=zeros(1,n);

t480_res_eco_li=zeros(1,n);

t480_int_eco_li=zeros(1,n);

t480_total_eco=zeros(1,n);

%number of SIR ecoli at t=480 (start of CTC);

%finding prop_xx_eco value steady state before CTC;

prop_res_ss_preCTC=zeros(1,n);

prop_int_ss_preCTC=zeros(1,n);

prop_susc_ss_preCTC=zeros(1,n);

prop_res_ss_CTC=zeros(1,n);

prop_int_ss_CTC=zeros(1,n);

prop_susc_ss_CTC=zeros(1,n);

res_pre_CTC_change=zeros(1,n);

int_pre_CTC_change=zeros(1,n);

susc_pre_CTC_change=zeros(1,n);

res_pre_post_change=zeros(1,n);

int_pre_post_change=zeros(1,n);

susc_pre_post_change=zeros(1,n);

ctc_li_conc=zeros(time/dt,n);

ctc_pl_conc=zeros(time/dt,n);

ctc_manure_conc=zeros(time/dt,n);

%ctc concentration in LI and plasma and manure;

ctc_st_amt=zeros(time/dt,n);

ctc_upsi_amt=zeros(time/dt,n);

ctc_rtsi_amt=zeros(time/dt,n);

ctc_tis_amt=zeros(time/dt,n);

%ctc absolute amount;

%Ex (PD effect) storage arrays;

Er_storage=zeros(time/dt,n);

Ei_storage=zeros(time/dt,n);

Es_storage=zeros(time/dt,n);

res_eco_growth=zeros(time/dt,n);

res_eco_outflow=zeros(time/dt,n);

res_eco_inflow=zeros(time/dt,n);

res_eco_cat_pop=zeros(time/dt,n);

%see tables for parameter definitions;

%E coli population inter-individual stochasticity;

if POPSTOCHA==0

Nmax=repmat(10^5.5,1,n);

pop=Nmax/2;

pr=repmat(0.35,1,n);

pi=repmat(0.15,1,n);

ps=repmat(0.50,1,n);

r=repmat(0.17,1,n);

bsi=repmat(0.002,1,n);

bsr=repmat(0.001569317647,1,n);

bir=repmat(0.0001452,1,n);

lamda_in=repmat(0.005,1,n);

lamda_out=repmat(0.01,1,n);

alpha=repmat(0.02,1,n);

start_r=repmat(0.378,1,n);

start_i=repmat(0.164,1,n);

start_s=repmat(0.458,1,n);

else

Nmax_dist=makedist('Weibull',14.03,20.32);

Nmax_trunc=truncate(Nmax_dist,3+7.59,8+7.59); %truncate (3,8)

Nmax_exp=random(Nmax_trunc,1,n)-7.59; %shift by 7.59

Nmax=10.^Nmax_exp;

% Nmax=10.^(wblrnd(14.03,20.32,1,n)-7.59);

pop=Nmax*unifrnd(.1,.9);

pr=unifrnd(0.16,0.61,1,n);

pi=unifrnd(0.02,0.15,1,n);

ps=1-pr-pi;

r=unifrnd(0.05,0.5,1,n);

bsi=10.^(gamrnd(94.17,0.16,1,n)-22.57);

bsr=10.^(gamrnd(94.17,0.16,1,n)-22.57);

bir=10.^(gamrnd(94.17,0.16,1,n)-22.57);

lamda_in=unifrnd(0.001,0.01,1,n);

lamda_out=unifrnd(0.01,0.02,1,n);

alpha=unifrnd(0,0.03,1,n);

start_r=unifrnd(0.16,0.61,1,n);

start_i=unifrnd(0.02,0.15,1,n);

start_s=1-start_r-start_i;

end

%PD inter-individual stochasticity;

if PDSTOCHA==0

Emax=1;

Hr=repmat(7.6,1,n);

Hi=repmat(7.6,1,n);

Hs=repmat(2.2,1,n);

%EC50r=repmat(47.2,1,n);

%EC50i=repmat(4.3,1,n);

%EC50s=repmat(0.2,1,n);

%if we use the anaerobic penalty to MIC:

anaerobe_s=repmat(-0.9,1,n);

anaerobe_i=repmat(-0.9,1,n);

anaerobe_r=repmat(-0.9,1,n);

MICr=repmat(48,1,n)+anaerobe_r;

MICi=repmat(8,1,n)+anaerobe_i;

MICs=repmat(4,1,n)+anaerobe_s;

EC50r=2.^(-1.24+1.09*log2(MICr));

EC50i=2.^(-1.24+1.09*log2(MICi));

EC50s=2.^(-1.24+1.09*log2(MICs));

else

Emax=1;

Hr=unifrnd(6.42,10,1,n);

Hi=unifrnd(5.71,9.53,1,n);

Hs=unifrnd(1.62,2.23,1,n);

%EC50r=repmat(47.2,1,n);

%EC50i=repmat(4.3,1,n);

%EC50s=repmat(0.2,1,n);

%if we use the anaerobic penalty to MIC:

%anaerobe_s=unifrnd(-1.3,0,1,n);

%anaerobe_i=unifrnd(-1.3,0,1,n);

%anaerobe_r=unifrnd(-1.3,0,1,n);

%MICr=unifrnd(16,64,1,n)+anaerobe_r;

%MICi=unifrnd(4,16,1,n)+anaerobe_i;

%MICs=abs(unifrnd(0,4,1,n)+anaerobe_s);

MICr=unifrnd(16-1.3,128,1,n);

MICi=unifrnd(4-1.3,16,1,n);

MICs=unifrnd(0,4,1,n);

EC50r=2.^(-1.24+1.09*log2(MICr));

EC50i=2.^(-1.24+1.09*log2(MICi));

EC50s=2.^(-1.24+1.09*log2(MICs));

end

if PKSTOCHA==0

delta=repmat(0.0333,1,n);

gamma_s=repmat(0.0715,1,n);

gamma_uppersi=repmat(0.333,1,n);

gamma_restsi=repmat(0.133,1,n);

gamma_li=repmat(0.133,1,n);

%eta_si=repmat(0.79,1,n);

eta_li=repmat(0.79,1,n);

ka=repmat(0.0478,1,n);

kpt=repmat(0.75,1,n);

ktp=repmat(0.162,1,n);

ke=repmat(1.14,1,n);

Eb=repmat(0.515,1,n);

Eu=1-Eb;

Vp=repmat(57*bw/1000,1,n);

Vli=repmat(11,1,n);

else

delta=betarnd(0.54,37.4,1,n);

gamma_s=unifrnd(0.0535,0.0895,1,n);

gamma_uppersi=unifrnd(0.25,0.416,1,n);

gamma_restsi=unifrnd(.1,.166,1,n);

gamma_li=unifrnd(0.1,0.166,1,n);

%eta_si=unifrnd(0.69,0.89,1,n);

eta_li=unifrnd(0.69,0.89,1,n);

ka=repmat(0.0478,1,n);

kpt=repmat(0.75,1,n);

ktp=repmat(0.162,1,n);

ke=repmat(1.14,1,n);

Eb=unifrnd(0.39,0.64,1,n);

Eu=1-Eb;

Vp=repmat(57*bw/1000,1,n);

Vli=unifrnd(6,22,1,n);

end

for j=1:n

%PK compartments that are overwritten each simulation;

CTC=zeros(time/dt,6); %1-s,2-upper_si,3-rest_si,4-pl,5-tis,6-li

T=zeros(time/dt,1);

CTC_feed=zeros(time/dt,1);

manure_ctc_conc=zeros(time/dt,1);

%E coli compartments that are overwritten each simulation;

pop_ecoli=zeros(time/dt,1);

susc_ecoli=zeros(time/dt,1);

resist_ecoli=zeros(time/dt,1);

int_ecoli=zeros(time/dt,1);

faeces=zeros(time/dt,1);

decay=zeros(time/dt,2);

%Initialization;

CTC(1,1)=0;

CTC(1,2)=0;

CTC(1,3)=0;

CTC(1,4)=0;

CTC(1,5)=0;

CTC(1,6)=0;

CTC_feed(1,1)=0;

manure_ctc_conc(1,1)=0;

susc_ecoli(1,1)=start_s(1,j)*pop(1,j);

resist_ecoli(1,1)=start_r(1,j)*pop(1,j);

int_ecoli(1,1)=start_i(1,j)*pop(1,j);

%starting amounts of SIR e coli;

pop_ecoli(1,1)=susc_ecoli(1,1)+resist_ecoli(1,1)+int_ecoli(1,1);

%starting values of stochastic arrays;

res_eco_li(1,j)=resist_ecoli(1,1);

int_eco_li(1,j)=int_ecoli(1,1);

susc_eco_li(1,j)=susc_ecoli(1,1);

prop_res_eco(1,j)=resist_ecoli(1,1)/pop_ecoli(1,1);

prop_int_eco(1,j)=int_ecoli(1,1)/pop_ecoli(1,1);

prop_susc_eco(1,j)=susc_ecoli(1,1)/pop_ecoli(1,1);

total_eco(1,j)=pop_ecoli(1,1);

ctc_li_conc(1,j)=CTC(1,6)/Vli(1,j);

ctc_pl_conc(1,j)=CTC(1,4)/Vp(1,j);

ctc_st_amt(1,j)=CTC(1,1);

ctc_upsi_amt(1,j)=CTC(1,2);

ctc_rtsi_amt(1,j)=CTC(1,3);

ctc_tis_amt(1,j)=CTC(1,5);

ctc_manure_conc(1,j)=manure_ctc_conc(1,1);

i=2;

while i<(time/dt)+1

T(i,1)=T(i-1,1)+dt;

% T (time) variable is in increments of 0.1, units of hours;

if T(i,1)>am_start %start CTC

if (mod(T(i,1),24) < 12)

if METHODE==0

CTCf=0;

else

if METHODE==1

if (T(i,1)<(adc_time+am_start))

CTCf=70/12; %ARLA dosage

else

CTCf=0;

end

elseif METHODE==2

if (T(i,1)<(adc_time+am_start))

CTCf=350/12; %ADC dosage

else

CTCf=0;

end

else

if (T(i,1)<(adt_time+am_start))

CTCf=22*bw/12; %ADT dosage

else

CTCf=0;

end

end

end

else

CTCf=0;

end

else

CTCf=0;

end

CTC_feed(i,1)=CTCf;

%intra-individual random components;

%st-stomach; in-income; po-degradation; up-upper SI; pl-plasma; re-rest SI; li-large intestine; ti-tissue; ou-outcome;

if INTRASTOCHA==0

rand_u=0;

rand_b=0;

rand_stin=0;

rand_stpo=0;

rand_stup=0;

rand_uppo=0;

rand_uppl=0;

rand_upre=0;

rand_repo=0;

rand_reli=0;

rand_plpo=0;

rand_plti=0;

rand_tipl=0;

rand_tipo=0;

rand_liou=0;

rand_lipo=0;

else

rand_u=normrnd(0,1);

rand_b=normrnd(0,1);

rand_stin=normrnd(0,1);

rand_stpo=normrnd(0,1);

rand_stup=normrnd(0,1);

rand_uppo=normrnd(0,1);

rand_uppl=normrnd(0,1);

rand_upre=normrnd(0,1);

rand_repo=normrnd(0,1);

rand_reli=normrnd(0,1);

rand_plpo=normrnd(0,1);

rand_plti=normrnd(0,1);

rand_tipl=normrnd(0,1);

rand_tipo=normrnd(0,1);

rand_liou=normrnd(0,1);

rand_lipo=normrnd(0,1);

end

%bile and urine CTC;

B=ke(1,j)*Eb(1,j)*CTC(i-1,4)+(ke(1,j)*Eb(1,j)*CTC(i-1,4)/dt)^(1/2)*rand_b;

U=ke(1,j)*Eu(1,j)*CTC(i-1,4)+(ke(1,j)*Eu(1,j)*CTC(i-1,4)/dt)^(1/2)*rand_u;

%CTC flows in and out of each intestinal compartment;

stomach_income=CTCf+(CTCf/dt)^(1/2)*rand_stin;

stomach_deg=-(delta(1,j)*CTC(i-1,1)+(delta(1,j)*CTC(i-1,1)/dt)^(1/2)*rand_stpo);

stom_uppersi=gamma_s(1,j)*CTC(i-1,1)+(gamma_s(1,j)*CTC(i-1,1)/dt)^(1/2)*rand_stup;

uppersi_deg=-(delta(1,j)*CTC(i-1,2)+(delta(1,j)*CTC(i-1,2)/dt)^(1/2)*rand_uppo);

uppersi_plasma=ka(1,j)*CTC(i-1,2)+(ka(1,j)*CTC(i-1,2)/dt)^(1/2)*rand_uppl;

uppersi_restsi=gamma_uppersi(1,j)*CTC(i-1,2)+(gamma_uppersi(1,j)*CTC(i-1,2)/dt)^(1/2)*rand_upre;

restsi_deg=-(delta(1,j)*CTC(i-1,3)+(delta(1,j)*CTC(i-1,3)/dt)^(1/2)*rand_repo);

restsi_li=gamma_restsi(1,j)*CTC(i-1,3)+(gamma_restsi(1,j)*CTC(i-1,3)/dt)^(1/2)*rand_reli;

plasma_deg=-(delta(1,j)*CTC(i-1,4)+(delta(1,j)*CTC(i-1,4)/dt)^(1/2)*rand_plpo);

plasma_tissue=kpt(1,j)*CTC(i-1,4)+(kpt(1,j)*CTC(i-1,4)/dt)^(1/2)*rand_plti;

tissue_plasma=ktp(1,j)*CTC(i-1,5)+(ktp(1,j)*CTC(i-1,5)/dt)^(1/2)*rand_tipl;

tissue_deg=-(delta(1,j)*CTC(i-1,5)+(delta(1,j)*CTC(i-1,5)/dt)^(1/2)*rand_tipo);

li_outcome=gamma_li(1,j)*CTC(i-1,6)+(gamma_li(1,j)*CTC(i-1,6)/dt)^(1/2)*rand_liou;

li_deg=-(delta(1,j)*CTC(i-1,6)+(delta(1,j)*CTC(i-1,6)/dt)^(1/2)*rand_lipo);

%Equations for CTC in each compartment

if CTC(i-1,1)+dt*(stomach_income+stomach_deg-stom_uppersi)>0

CTC(i,1)=CTC(i-1,1)+dt*(stomach_income+stomach_deg-stom_uppersi);

else

CTC(i,1)=0;

end

if CTC(i-1,2)+dt*(stom_uppersi+B+uppersi_deg-uppersi_plasma-uppersi_restsi)>0

CTC(i,2)=CTC(i-1,2)+dt*(stom_uppersi+B+uppersi_deg-uppersi_plasma-uppersi_restsi);

else

CTC(i,2)=0;

end

if CTC(i-1,3)+dt*(restsi_deg+uppersi_restsi-restsi_li)>0

CTC(i,3)=CTC(i-1,3)+dt*(restsi_deg+uppersi_restsi-restsi_li);

else

CTC(i,3)=0;

end

if CTC(i-1,4)+dt*(uppersi_plasma-B-U+plasma_deg-plasma_tissue+tissue_plasma)>0

CTC(i,4)=CTC(i-1,4)+dt*(uppersi_plasma-B-U+plasma_deg-plasma_tissue+tissue_plasma);

else

CTC(i,4)=0;

end

if CTC(i-1,5)+dt*(plasma_tissue+tissue_deg-tissue_plasma)>0

CTC(i,5)=CTC(i-1,5)+dt*(plasma_tissue+tissue_deg-tissue_plasma);

else

CTC(i,5)=0;

end

if CTC(i-1,6)+dt*(restsi_li+li_deg-li_outcome)>0

CTC(i,6)=CTC(i-1,6)+dt*(restsi_li+li_deg-li_outcome);

else

CTC(i,6)=0;

end

%12-30-16 new manure concentration;

manure_ctc_conc(i,1)=((li_outcome+U)/((0.001667*bw)+(0.00075*bw)))*(1-eta_li(1,j));

%above: 0.001667 is the defecation volume per kg bw per hr;

%PD equations;

Es=1-((Emax*((CTC(i-1,6)/Vli(1,j))*(1-eta_li(1,j))).^Hs(1,j))/((EC50s(1,j)).^Hs(1,j)+((CTC(i-1,6)/Vli(1,j))*(1-eta_li(1,j))).^Hs(1,j)));

Er=1-((Emax*((CTC(i-1,6)/Vli(1,j))*(1-eta_li(1,j))).^Hr(1,j))/((EC50r(1,j)).^Hr(1,j)+((CTC(i-1,6)/Vli(1,j))*(1-eta_li(1,j))).^Hr(1,j)));

Ei=1-((Emax*((CTC(i-1,6)/Vli(1,j))*(1-eta_li(1,j))).^Hi(1,j))/((EC50i(1,j)).^Hi(1,j)+((CTC(i-1,6)/Vli(1,j))*(1-eta_li(1,j))).^Hi(1,j)));

%E coli population

%intra-individual random components

%growth

if INTRASTOCHA==0

rand_g_c=0;

rand_s_g_c_1=0;

rand_s_g_c_2=0;

%g_c=growth coefficient;

rand_r_g_c_1=0;

rand_r_g_c_2=0;

rand_i_g_c_1=0;

rand_i_g_c_2=0;

rand_pl_si=0;

rand_pl_sr=0;

rand_pl_ir=0;

%pl=plasmid in the e coli population model;

else

rand_g_c=normrnd(0,1);

rand_s_g_c_1=normrnd(0,1);

rand_s_g_c_2=normrnd(0,1);

rand_r_g_c_1=normrnd(0,1);

rand_r_g_c_2=normrnd(0,1);

rand_i_g_c_1=normrnd(0,1);

rand_i_g_c_2=normrnd(0,1);

rand_pl_si=normrnd(0,1);

rand_pl_sr=normrnd(0,1);

rand_pl_ir=normrnd(0,1);

end

%new plasmid transfer equations;

if pop_ecoli(i-1,1)>0

plasmid_transfert_si=bsi(1,j)*susc_ecoli(i-1,1)*int_ecoli(i-1,1)/(pop_ecoli(i-1,1))+abs((bsi(1,j)*susc_ecoli(i-1,1)*int_ecoli(i-1,1)/pop_ecoli(i-1,1))/dt)^(1/2)*rand_pl_si;

else

plasmid_transfert_si=0;

end

if pop_ecoli(i-1,1)>0

plasmid_transfert_sr=bsr(1,j)*susc_ecoli(i-1,1)*resist_ecoli(i-1,1)/(pop_ecoli(i-1,1))+abs((bsr(1,j)*susc_ecoli(i-1,1)*resist_ecoli(i-1,1)/pop_ecoli(i-1,1))/dt)^(1/2)*rand_pl_sr;

else

plasmid_transfert_sr=0;

end

if pop_ecoli(i-1,1)>0

plasmid_transfert_ir=bir(1,j)*int_ecoli(i-1,1)*resist_ecoli(i-1,1)/( pop_ecoli(i-1,1))+abs((bir(1,j)*int_ecoli(i-1,1)*resist_ecoli(i-1,1)/pop_ecoli(i-1,1))*dt)^(1/2)*rand_pl_ir;

else

plasmid_transfert_ir=0;

end

%e coli pop growth in cattle large intestine

susc_cat_population=r(1,j)*(1-pop_ecoli(i-1,1)/(Nmax(1,j)))*Es*susc_ecoli(i-1,1)+abs((r(1,j)*(1-pop_ecoli(i-1,1)/(Nmax(1,j)))*abs(Es)*susc_ecoli(i-1,1))/dt)^(1/2)*rand_s_g_c_1+ps(1,j)*lamda_in(1,j)*pop_ecoli(i-1,1)+abs((ps(1,j)*lamda_in(1,j)*pop_ecoli(i-1,1)/dt))^(1/2)*rand_g_c-lamda_out(1,j)*susc_ecoli(i-1,1)-abs((lamda_out(1,j)*susc_ecoli(i-1,1)/dt))^(1/2)*rand_s_g_c_2;

res_cat_population=r(1,j)*(1-alpha(1,j))*(1-pop_ecoli(i-1,1)/(Nmax(1,j)))*Er*resist_ecoli(i-1,1)+abs((r(1,j)*(1-alpha(1,j))*(1-pop_ecoli(i-1,1)/(Nmax(1,j)))*abs(Er)*resist_ecoli(i-1,1)/dt))^(1/2)*rand_r_g_c_1+pr(1,j)*lamda_in(1,j)*pop_ecoli(i-1,1)+abs((pr(1,j)*lamda_in(1,j)*pop_ecoli(i-1,1)/dt))^(1/2)*rand_g_c-lamda_out(1,j)*resist_ecoli(i-1,1)-abs((lamda_out(1,j)*resist_ecoli(i-1,1)/dt))^(1/2)*rand_r_g_c_2;

int_cat_population=r(1,j)*(1-alpha(1,j))*(1-pop_ecoli(i-1,1)/(Nmax(1,j)))*Ei*int_ecoli(i-1,1)+abs((r(1,j)*(1-alpha(1,j))*(1-pop_ecoli(i-1,1)/(Nmax(1,j)))*abs(Ei)*int_ecoli(i-1,1)/dt))^(1/2)*rand_i_g_c_1+pi(1,j)*lamda_in(1,j)*pop_ecoli(i-1,1)+abs((pi(1,j)*lamda_in(1,j)*pop_ecoli(i-1,1)/dt))^(1/2)*rand_g_c-lamda_out(1,j)*int_ecoli(i-1,1)-abs((lamda_out(1,j)*int_ecoli(i-1,1)/dt))^(1/2)*rand_i_g_c_2;

%record the growth, inflow and outflow of resistant bacteria. Used to check for errors and inappropriate model behavior;

res_eco_growth(i,1)=r(1,j)*(1-alpha(1,j))*(1-pop_ecoli(i-1,1)/(Nmax(1,j)))*Er*resist_ecoli(i-1,1);

res_eco_inflow(i,1)=pr(1,j)*lamda_in(1,j)*pop_ecoli(i-1,1);

res_eco_outflow(i,1)=-lamda_out(1,j)*resist_ecoli(i-1,1);

res_eco_cat_pop(i,1)=res_cat_population;

%Equations for e coli population

susc_ecoli(i,1)=susc_ecoli(i-1,1)+dt*(susc_cat_population-plasmid_transfert_sr-plasmid_transfert_si);

resist_ecoli(i,1)=resist_ecoli(i-1,1)+dt*(res_cat_population+plasmid_transfert_sr+plasmid_transfert_ir);

int_ecoli(i,1)=int_ecoli(i-1,1)+dt*(int_cat_population+plasmid_transfert_si-plasmid_transfert_ir);

pop_ecoli(i,1)=susc_ecoli(i,1)+resist_ecoli(i,1)+int_ecoli(i,1);

%store data in arrays;

res_eco_li(i,j)=resist_ecoli(i,1);

int_eco_li(i,j)=int_ecoli(i,1);

susc_eco_li(i,j)=susc_ecoli(i,1);

prop_res_eco(i,j)=resist_ecoli(i,1)/pop_ecoli(i,1);

prop_int_eco(i,j)=int_ecoli(i,1)/pop_ecoli(i,1);

prop_susc_eco(i,j)=susc_ecoli(i,1)/pop_ecoli(i,1);

total_eco(i,j)=pop_ecoli(i,1);

ctc_li_conc(i,j)=(CTC(i,6)/Vli(1,j))*(1-eta_li(1,j));

ctc_pl_conc(i,j)=CTC(i,4)/Vp(1,j);

ctc_manure_conc(i,j)=manure_ctc_conc(i,1);

ctc_st_amt(i,j)=CTC(i,1);

ctc_upsi_amt(i,j)=CTC(i,2);

ctc_rtsi_amt(i,j)=CTC(i,3);

ctc_tis_amt(i,j)=CTC(i,5);

%Ex storage array;

Er_storage(i,j)=Er;

Ei_storage(i,j)=Ei;

Es_storage(i,j)=Es;

i=i+1;

end

%calculations of SIR proportions and amounts at different time points;

%amount of e coli at 48 hours.;

t480_susc_eco_li(1,j)=susc_eco_li(am_start*10,j);

t480_res_eco_li(1,j)=res_eco_li(am_start*10,j);

t480_int_eco_li(1,j)=int_eco_li(am_start*10,j);

t480_total_eco(1,j)=total_eco(am_start*10,j);

%SIR in last 6 hours before tx;

prop_res_ss_preCTC(1,j)=mean(prop_res_eco((am_start-6)*10:am_start*10,j));

prop_int_ss_preCTC(1,j)=mean(prop_int_eco((am_start-6)*10:am_start*10,j));

prop_susc_ss_preCTC(1,j)=mean(prop_susc_eco((am_start-6)*10:am_start*10,j));

%to find the average during tx;

if METHODE==3 %ADT

tx_end_time=am_start+adt_time;

%for ADT, average between tx start (day 2) and day 10 (3 days after tx

%ends), which is when resistance peaks and LI CTC conc drops to almost 0;

prop_res_ss_CTC(1,j)=mean(prop_res_eco(am_start*10:(tx_end_time+72)*10,j));

prop_int_ss_CTC(1,j)=mean(prop_int_eco(am_start*10:(tx_end_time+72)*10,j));

prop_susc_ss_CTC(1,j)=mean(prop_susc_eco(am_start*10:(tx_end_time+72)*10,j));

%max during tx (see time frame above). added 5d to end of tx just to be sure got the max for every simulation since in ADT max occurs after treatment ends;

[prop_res_max, prop_res_time_max]=max(prop_res_eco(am_start*10:(tx_end_time+120)*10,:));

[prop_int_max, prop_int_time_max]=max(prop_int_eco(am_start*10:(tx_end_time+120)*10,:));

[prop_susc_max, prop_susc_time_max]=max(prop_susc_eco(am_start*10:(tx_end_time+120)*10,:));

%to find the average number of e coli during tx;

total_eco_ss_CTC(1,j)=mean(total_eco(am_start*10:(tx_end_time+72)*10,j));

else %ARLA, ADC or no CTC

tx_end_time=am_start+adc_time;

%for ADC and ARLA, an average also over 7 days (as above) but at the end of tx.;

prop_res_ss_CTC(1,j)=mean(prop_res_eco((tx_end_time-(7*24))*10:(tx_end_time)*10,j));

prop_int_ss_CTC(1,j)=mean(prop_int_eco((tx_end_time-(7*24))*10:(tx_end_time)*10,j));

prop_susc_ss_CTC(1,j)=mean(prop_susc_eco((tx_end_time-(7*24))*10:(tx_end_time)*10,j));

%max during tx;

[prop_res_max, prop_res_time_max]=max(prop_res_eco(am_start*10:(tx_end_time+120)*10,:));

[prop_int_max, prop_int_time_max]=max(prop_int_eco(am_start*10:(tx_end_time+120)*10,:));

[prop_susc_max, prop_susc_time_max]=max(prop_susc_eco(am_start*10:(tx_end_time+120)*10,:));

%to find the average number of e coli during tx (last 7 days of tx);

total_eco_ss_CTC(1,j)=mean(total_eco((tx_end_time-(7*24))*10:(tx_end_time)*10,j));

end

%proportion SIR averaged over the last 24 hours;

prop_susc_ss_postCTC(1,j)=mean(prop_susc_eco((time-24)*10:time*10,j));

prop_int_ss_postCTC(1,j)=mean(prop_int_eco((time-24)*10:time*10,j));

prop_res_ss_postCTC(1,j)=mean(prop_res_eco((time-24)*10:time*10,j));

% e coli before treatment

total_eco_ss_preCTC(1,j)=mean(total_eco((am_start-6)*10:am_start*10,j));

%e coli after tx;

total_eco_ss_postCTC(1,j)=mean(total_eco((time-24)*10:time*10,j));

end
